# Changes in proportions of Cesarean section before and during the COVID‐19 pandemic in Japan

**DOI:** 10.1111/jog.16370

**Published:** 2025-07-10

**Authors:** Kensuke Shimada, Jun Komiyama, Takehiro Sugiyama, Shin Jung‐Ho, Tomomi Kihara, Rie Masuda, Susumu Kunisawa, Masao Iwagami, Isao Muraki, Yuichi Imanaka, Hiroyasu Iso, Nanako Tamiya

**Affiliations:** ^1^ Graduate School of Comprehensive Human Sciences University of Tsukuba Ibaraki Japan; ^2^ Transrational Research Promotion Center, Tsukuba Clinical Research & Development Organization University of Tsukuba Ibaraki Japan; ^3^ Department of Anesthesiology University of Tsukuba Hospital Ibaraki Japan; ^4^ Department of Health Services Research, Institute of Medicine University of Tsukuba Ibaraki Japan; ^5^ Health Services Research and Development Center University of Tsukuba Ibaraki Japan; ^6^ Diabetes and Metabolism Information Center, National Institute of Global Health and Medicine Japan Institute for Health Security Tokyo Japan; ^7^ Institute for Global Health Policy Research, Bureau of Global Health Cooperation Japan Institute for Health Seculity Tokyo Japan; ^8^ Department of Healthcare Economics and Quality Management, Graduate School of Medicine Kyoto University Kyoto Japan; ^9^ Department of Public Health Medicine, Institute of Medicine University of Tsukuba Ibaraki Japan; ^10^ Department of Social and Environmental Medicine, Graduate School of Medicine Osaka University Osaka Japan; ^11^ Department of Health Security System, Center for Health Security, Graduate School of Medicine Kyoto University Kyoto Japan; ^12^ Center for Artificial Intelligence Research University of Tsukuba Ibaraki Japan; ^13^ Center for Cyber Medicine Research University of Tsukuba Ibaraki Japan

**Keywords:** Cesarean section, coronavirus disease 2019 (COVID‐19), Japan, National Database of Health Insurance Claims and Specific Health Checkups of Japan (NDB), mode of delivery

## Abstract

**Aim:**

After the coronavirus disease 2019 (COVID‐19) pandemic, Cesarean sections for COVID‐19‐positive cases were performed to reduce delivery time and thus control infection. This may have increased the proportion of Cesarean sections and affected many pregnant women in Japan; though this expected trend has not yet been quantified. This study examined changes in the proportions of Cesarean sections in Japan before and during the pandemic.

**Methods:**

This study retrospective observational study used the National Database of Health Insurance Claims and Specific Health Checkups of Japan and Vital Statistics from the National Statistical Surveys from April 2018 to October 2022. We compared proportions of Cesarean sections (total Cesarean sections/total live births) in Japan before and during the pandemic and by the COVID‐19 pandemic phase: pre‐COVID‐19 (April 2018 to December 2019), Wave 1 (January to May 2020), Wave 2 (June to October 2020), Wave 3 (November 2020 to February 2021), Wave 4 (March to June 2021), Wave 5 (July to December 2021), Wave 6 (January to June 2022), and Wave 7 (July to October 2022).

**Results:**

The proportion of Cesarean sections in Japan was 20.27% (317 241/1 564 912) before the pandemic and increased to 21.19% (486 172/2 294 488) during the pandemic. The highest proportion was in Wave 6 (22.14%), dropping to 21.27% in Wave 7.

**Conclusion:**

The overall proportion of Cesarean sections increased by 0.92% point during the COVID‐19 pandemic in Japan, possibly due in part to infection control measures. Verification and preparation are necessary to respond to future pandemics.

## INTRODUCTION

The coronavirus disease 2019 (COVID‐19) has had a profound impact on patients and healthcare systems.^1^, ^2^ Cardiovascular mortality and cancer progression have increased due to delayed diagnosis and treatment.^3^, ^4^, ^5^ Other impacts ranging from the supply of blood products to organ transplants have been reported.^6^, ^7^

During the COVID‐19 pandemic, maternal and fetal outcomes were reported to have deteriorated worldwide.^8^ There have also been reports of changes in the mode of delivery, including elective Cesarean sections indicated for COVID‐19 as part of institutional infection control and other purposes.^9^ Although the first two editions of the Japanese guidelines did not recommend Cesarean section for COVID‐19 infection (March 5, 2020, and March 20, 2020), from the third edition on April 7, 2020, the following was included in the guidelines: “COVID‐19 infection alone is an indication for Cesarean section. However, Cesarean section might be considered to shorten delivery management time in light of the medical resources that can be allocated to infection control at the facility and the general condition of the pregnant woman, including pneumonia.”^10^ Therefore, it is possible that the COVID‐19 pandemic affected the mode of delivery in Japan. A study using data from registered facilities has suggested changes in delivery method trends in Japan.^11^ However, no studies have investigated changes in the proportions of Cesarean sections using nationwide data in Japan.

An unplanned Cesarean section can impact a person's family plans and later life.^12^, ^13^ The purpose of this study was to determine the changes in the proportions of Cesarean sections before and during the COVID‐19 pandemic in Japan, as well as regional differences. This study utilized the National Database of Health Insurance Claims and Specific Health Checkup of Japan (NDB) to comprehensively calculate the number of Cesarean sections in Japan.^14^

## METHODS

This was a retrospective descriptive study. Ethical approval for this study (no. 1833) was obtained from the Institutional Review Board of the University of Tsukuba on November 25, 2022. The requirement for informed consent was waived owing to the anonymity of data.

### Data sources and variables

The data we used are summarized in Table 1. Since the data sources were compiled independently within each dataset, no consistent inclusion or exclusion criteria were established.

**TABLE 1:** Details of data sources.

Data source	Variables
NDB (Ministry of Health, Labour and Welfare, Japan)	Number of Cesarean sections (monthly, by prefecture)
	Number of emergency Cesarean sections (monthly, by prefecture)
	Number of COVID‐19‐positive Cesarean sections (monthly, by prefecture)
	Number of COVID‐19‐positive emergency Cesarean sections (monthly, by prefecture)
Vital statistics (Ministry of Health, Labour and Welfare, Japan)	Number of live births (monthly, by prefecture)
Visualizing the data: information on COVID‐19 infections (Ministry of Health, Labour and Welfare, Japan)	Number of newly confirmed COVID‐19 cases (daily, by prefecture)
Population estimates (Statistical Bureau, Ministry of Internal Affairs and Communications, Japan)	Japanese population (monthly, Japan as a whole)
	Japanese population (yearly, by prefecture)
System of social and demographic statistics (Statistical Bureau, Ministry of Internal Affairs and Communications)	Population per 1 km^2^ of inhabitable area (yearly, by prefecture)
Physician, dentist and pharmacist statistics (Ministry of Health, Labour and Welfare, Japan)	Number of physicians with board certification in obstetrics and gynecology (every 2 years, by prefecture)
	Number of physicians with board certification in obstetrics and gynecology per 100 000 women aged 15–49 years (every 2 years, by prefecture)

*Note*: COVID‐19, coronavirus disease 2019; NDB, National Database of Health Insurance Claims and Specific Health Checkup of Japan.

### National Database of Health Insurance Claims and Specific Health Checkup of Japan

In Japan, all residents must join the health insurance system as a national health coverage system.^15^ The NDB database includes insurance claims for services such as medical examinations from nearly all hospitals and clinics in Japan.^15^, ^16^ From this database, we counted Cesarean section claims with or without the disease code of COVID‐19 in the same month of the Cesarean section. As a rule for using this database, data with aggregate values of 1–9 cannot be disclosed; therefore, these values are labeled “1–9” in tables, and, for illustrative purposes, the number 5 was used instead. The data used in this study were extracted from the NDB to investigate changes in the number of surgical and other medical procedures performed before and during the COVID‐19 pandemic. Accordingly, the data did not include information on diseases other than COVID‐19.

### Other data

For the number of live births, the “Vital Statistics (Ministry of Health, Labour and Welfare, Japan)” was used (accessed January 12, 2024).^17^ For the number of newly confirmed COVID‐19 cases, the “Visualizing the data: information on COVID‐19 infections (Ministry of Health, Labour and Welfare, Japan)” was used (accessed February 16, 2023).^18^ For calculating the Japanese population, the “Population Estimates (Statistical Bureau, Ministry of Internal Affairs and Communications, Japan)” was used (accessed January 12, 2024).^19^ For the population per 1 km^2^ of inhabitable area, the “System of Social and Demographic Statistics (Statistical Bureau, Ministry of Internal Affairs and Communications)” was used (accessed April 5, 2024).^20^ To calculate the number of obstetricians per population, the “Physician, Dentist and Pharmacist Statistics (Ministry of Health, Labour and Welfare, Japan)” was used (accessed May 15, 2024).^21^ The above data listed in this section are publicly available.

### Definition of the COVID‐19 pandemic phase

The first case of COVID‐19 infection in Japan was confirmed in January 2020; therefore, the period from January 2020 to October 2022 is defined as the COVID‐19 period, while the period before January 2020 is defined as the pre‐COVID‐19 period. In Japan, there were waves of COVID‐19 prevalence, each assigned a number. For the definitions of the waves, since the data we used were organized by months, we considered the months in which one wave approximately begins to the month in which the next wave approximately begins to be one wave of infection. Specifically, we used the following wave definitions: pre‐COVID‐19 (April 2018 to December 2019), Wave 1 (January to May 2020), Wave 2 (June to October 2020), Wave 3 (November 2020 to February 2021), Wave 4 (March to June 2021), Wave 5 (July to December 2021), Wave 6 (January to June 2022), and Wave 7 (July to October 2022).^22^, ^23^, ^24^, ^25^

### Measurements and basic calculations

Data from April 1, 2018, to October 31, 2022, were used. The number of Cesarean sections, the number of COVID‐19‐positive Cesarean sections, the number of live births, the proportions of Cesarean sections, the proportion of COVID‐19‐positive Cesarean sections, COVID‐19 incidences, and differences in the proportion of Cesarean sections were defined and calculated as indicated below in the manuscript.

#### Number of Cesarean sections

The number of Cesarean sections was calculated by counting the instances of Cesarean sections (billing code: “150222110” or “150222210”) for each prefecture by month. Among these, emergency Cesarean sections were specifically identified by counting the billing code “150222110.”

#### Number of COVID‐19‐positive Cesarean sections

The number of COVID‐19‐positive Cesarean sections was determined by counting the Cesarean sections for which COVID‐19 disease was registered on the claim in the same month as the Cesarean section claim. It was not possible to analyze the data on a daily or weekly basis. COVID‐19 was defined by the International Classification of Diseases, 10th Edition (ICD‐10) as follows: “B342,” “U071,” “U072,” “U08,” “U089,” “U09,” “U099,” “U10,” “U109.” Calculations were made regardless of the presence of suspicious flags, as a Cesarean section may have been performed on pregnant women suspected of having COVID‐19. The suspicious flag is used to record the disease name at the time of a tentative diagnosis when the name of the disease has not yet been confirmed. Additionally, the number of COVID‐19‐positive Cesarean sections, excluding suspicious flags, was also calculated to account for confirmed cases supplementally. Note that, because we used a dataset derived from the NDB to conduct the study on COVID‐19, the available variables were limited, and we could not include disease codes other than COVID‐19.

#### Number of live births

We used the number of live births from Vital Statistics. Births occurring in foreign countries were excluded from the total to conduct prefecture‐by‐prefecture comparisons later.

#### Proportions of Cesarean sections

The proportion of Cesarean section was defined as

$$\begin{array}{l} \text{Proportion of Cesarean section}\left(\%\right)\\ \;=\frac{\text{Number of Cesarean sections}}{\text{Number of live births}}\times 100\%. \end{array}$$

#### Proportion of COVID‐19‐positive Cesarean sections

The proportion of COVID‐19‐positive Cesarean section was defined as

$$\begin{array}{l} \text{Proportion of COVID}‐19‐\text{positive Cesarean section}\left(\%\right)\\ \;=\begin{array}{l} \frac{\text{Number of COVID}‐19‐\text{positive Cesarean sections}}{\text{Number of live births}}\times 100\%. \end{array} \end{array}$$

#### Proportion of emergency among Cesarean sections

The Proportion of emergency among Cesarean section was defined as

$$\begin{array}{l} \text{Proportion of emergency among Cesarean section}\left(\%\right)\\ \;=\frac{\text{Number of emergency Cesarean sections}}{\text{Number of Cesarean sections}}\times 100\%. \end{array}$$

#### Proportion of COVID‐19‐positive emergency among Cesarean sections

The Proportion of COVID‐19‐positive emergency among Cesarean section was defined as

$$\begin{array}{l} \text{Proportion of COVID}‐19‐\text{positive emergency among Cesarean section}\left(\%\right)\\ \;=\begin{array}{l} \frac{\text{Number of COVID}‐19‐\text{positive emergency Cesarean sections}}{\text{Number of Cesarean sections}}\times 100\%. \end{array} \end{array}$$

#### COVID‐19 incidence

The COVID‐19 incidence was defined as

$$\text{COVID}‐19\;\text{incidence}=\frac{\text{Number of COVID}‐19‐\text{positive cases}\;\mathrm{on}\;\text{the month in �Japan}}{\text{�Japanese population}\;\mathrm{on}\;\text{the month}}.$$

#### Difference in the proportion of Cesarean sections

The difference in the proportion of Cesarean section was defined as

$$\begin{array}{l} \text{Difference in proportion of Cesarean section}\left(\%\right)\\ \;=\begin{array}{l} \text{Proportion of Cesarean �section�in the interest}\\ ‐\;\text{period}\left(\%\right)-\text{proportion of Cesarean �section�in the}\;\mathrm{pre}\\ ‐\;\text{COVID}‐19\left(\%\right). \end{array} \end{array}$$

### Statistical analysis

For the trends in Cesarean sections and the COVID‐19 pandemic in Japan as a whole, the proportion of Cesarean sections, the proportion of COVID‐19‐positive Cesarean sections, the proportion of emergency among Cesarean sections, the proportion of COVID‐19‐positive emergency among Cesarean sections, and the COVID‐19 incidence were visualized and summarized by month and COVID‐19 pandemic phase. Additionally, a contingency table was used to examine whether the proportion of emergency Cesarean sections during the COVID‐19 period was higher among COVID‐19‐positive pregnant women than among pregnant women without COVID‐19 flags.

To further assess the robustness of our findings, we conducted an additional statistical analysis using Poisson regression with robust standard errors to evaluate changes in the proportion of Cesarean section before and during the COVID‐19 pandemic in Japan as a whole. In this model, the monthly number of Cesarean sections was used as the dependent variable, and the total number of births per month was included as an offset term to account for variations in delivery volume. The pandemic period was modeled as a binary exposure variable (pre‐COVID19 pandemic vs. COVID‐19 pandemic). Additionally, we adjusted for calendar month to account for potential seasonality. The regression was applied to the entire dataset, including all eligible deliveries.

Since the proportion of Cesarean section had not been compared nationwide after 2013, the differences in the proportion of Cesarean section were visualized as maps of the COVID‐19 pandemic phases.^26^ However, due to limitations in the available data, other factors potentially associated with the proportion of Cesarean section were not examined in this study.

Finally, to explore variables that might be associated with the difference in the proportion of Cesarean section between the pre‐COVID‐19 and COVID‐19 periods by prefecture, we selected COVID‐19 incidence, population density of inhabitable land area, the number of obstetricians and gynecologists per 100 000 women aged 15–49 years, and the number of births per obstetrician/gynecologist for each prefecture. The COVID‐19 incidence for each prefecture was calculated as follows:

$$\begin{array}{l} \text{COVID}‐19\;\text{incidence for each prefecture}\\ \;=\frac{\text{Number of COVID}‐19‐\text{positive cases for each prefecture from April�}\;1,2020\;\text{to October�}\;31,2022}{\text{�Japanese population for each prefecture}\;\mathrm{on}\;\text{October�}\;1,2020}. \end{array}$$

The population density of inhabitable land area was calculated using the mean of each year's data of the population density of inhabitable land area from 2018 to 2022. The number of obstetricians and gynecologists per population was determined by the average number of physicians with board certification in obstetrics and gynecology per 100 000 women aged 15–49 years in 2018, 2020, and 2022. The number of births per obstetrician/gynecologist was calculated as the average of the number of births in the fiscal years 2018, 2020, and 2022 (April to March of the following year) divided by the total number of physicians with board certification in obstetrics and gynecology in 2018, 2020, and 2022. The associations between these variables and the differences in the proportion of Cesarean sections were examined using scatter plots and correlation coefficients (Pearson's product–moment correlation).

R software (version 4.2.2) was used for all statistical analyses and figure creation. A *p*‐value <0.05 was considered to be statistically significant.

## RESULTS

### Trends in Cesarean sections and the COVID‐19 pandemic for Japan as a whole

**FIGURE 1:**
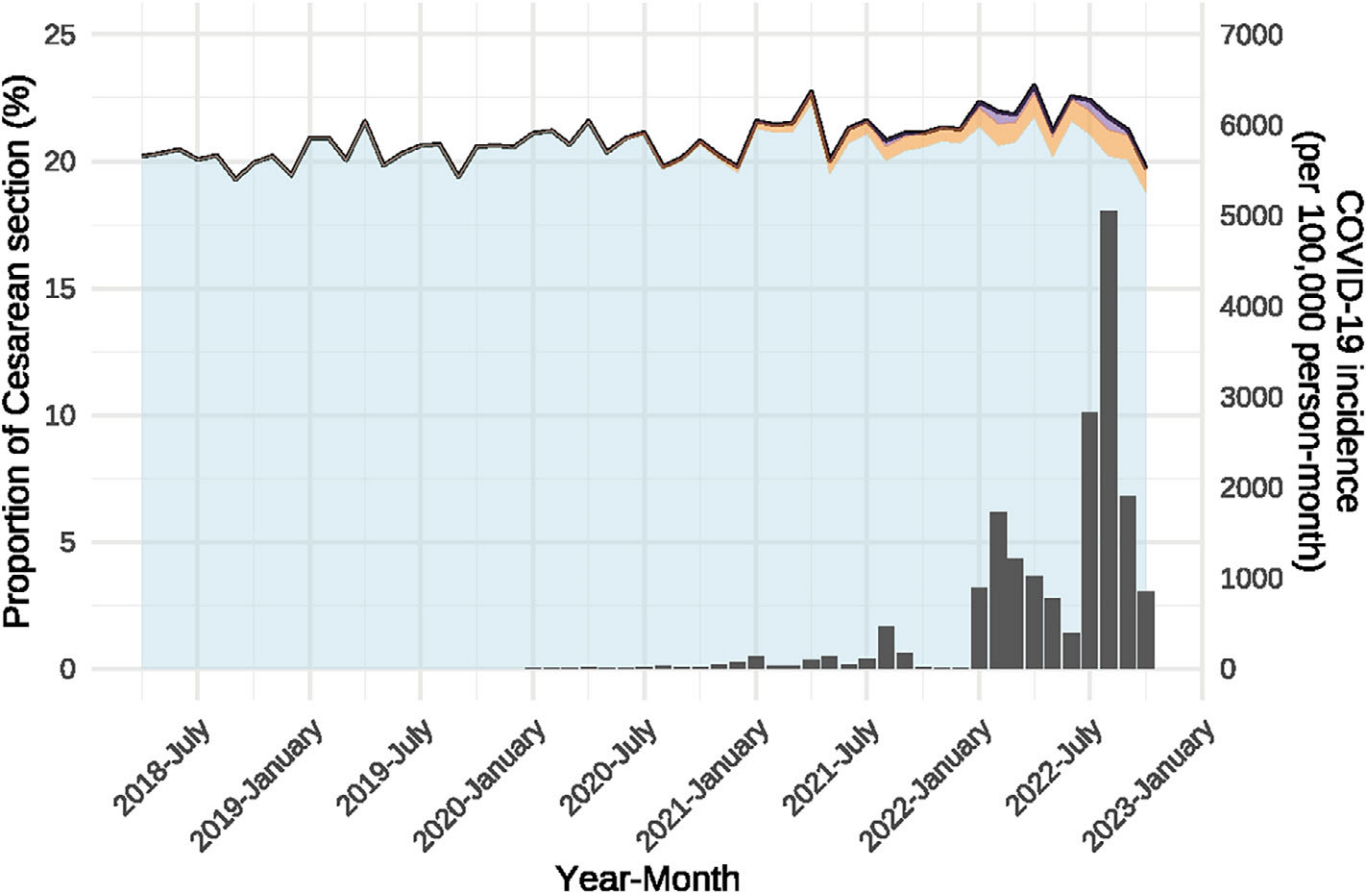
Proportion of Cesarean section and number of patients with COVID‐19 by month in Japan. The solid line represents the national proportion of the Cesarean section. The blue area indicates the Cesarean sections without the COVID‐19 disease code, the orange area indicates the COVID‐19‐positive Cesarean sections (with the suspicions flag), and the purple area indicates the COVID‐19‐positive Cesarean sections (excluding the suspicious flag). The bar chart shows the number of patients with COVID‐19.

**FIGURE 2:**
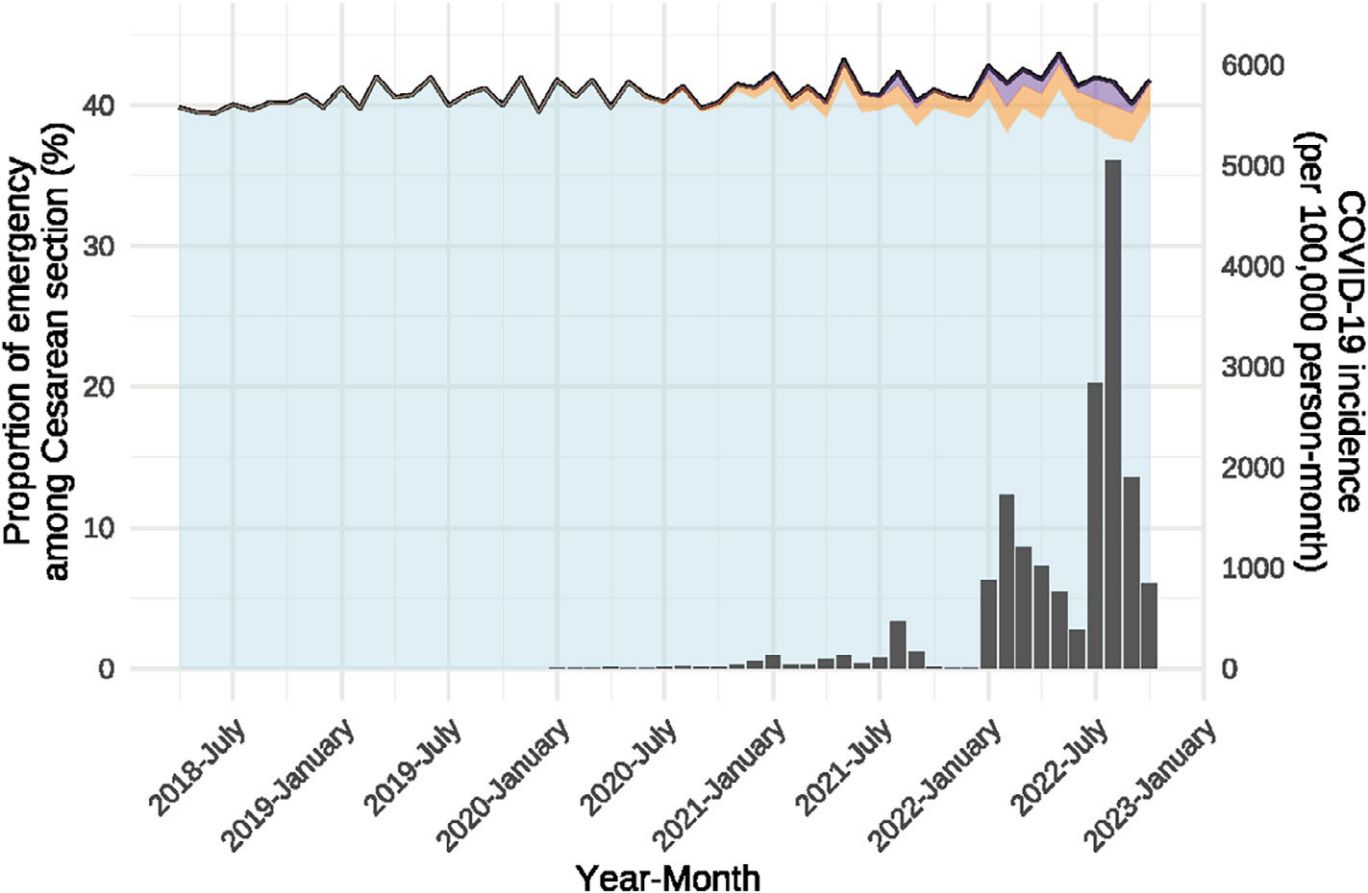
Proportion of emergency among Cesarean section and number of patients with COVID‐19 by month in Japan. The solid line represents the national proportion of emergency among Cesarean sections. The blue area indicates the emergency Cesarean sections without the COVID‐19 disease code, the orange area indicates the COVID‐19‐positive emergency Cesarean sections (with the suspicions flag), and the purple area indicates the COVID‐19‐positive emergency Cesarean sections (excluding the suspicious flag). The bar chart shows the number of patients with COVID‐19.

The trends in the proportions of Cesarean sections, the proportions of COVID‐19‐positive Cesarean sections, the proportions of emergencies among Cesarean sections, the proportions of COVID‐19‐positive emergencies among Cesarean sections, and COVID‐19 incidence in Japan are shown by month (Figures 1 and 2 and Files S1 and S2). From Figure 1, we can see that the proportion of Cesarean sections hovered around 20% before the COVID‐19 pandemic, but shortly after the pandemic began, the proportion plateaued at around 22%. Figure 2 shows that the proportion of emergency cases for Cesarean sections also increased from approximately 40% before the COVID‐19 pandemic to approximately 41% after the COVID‐19 pandemic began.

Table 2 presents these trends for the phases of the COVID‐19 pandemic. The proportion of Cesarean sections increased by 0.92% from 20.27% (317 241/1 564 912) in the pre‐COVID‐19 period to 21.19% (486 172/2 294 488) in the COVID‐19 period. The proportions of COVID‐19‐positive Cesarean sections were 0.54% (12 391/2 294 488) and 0.10% (2361/2 294 488) when excluding the suspicious flag, which was less than the difference in the proportions of the Cesarean sections between the pre‐COVID‐19 and COVID‐19 periods. A breakdown of the COVID‐19 periods showed that the increase in the proportion of Cesarean sections was greatest during the sixth wave at 22.14% (81 299/367 232), coinciding with a marked increase in the number of COVID‐19 cases nationwide. In the seventh wave, when the number of COVID‐19 cases was higher than that in the sixth wave, the increase was not as pronounced, at 21.27% (58 475/274 859). The proportions of emergencies among Cesarean sections also increased by 0.84%, from 40.41% (128 199/317 241) in the pre‐COVID‐19 period to 41.25% (200 561/486 172) in the COVID‐19 period. Similar to the proportion of Cesarean sections, the breakdown of the COVID‐19 periods also showed that the increase in the proportion of emergencies among Cesarean sections was greatest in the sixth wave, at 42.28% (34 376/81 299).

**TABLE 2:** Proportion of Cesarean section and other trends according to pandemic waves of infection.

Period	Pre‐COVID‐19 period	During COVID‐19 period							
		Wave 1	Wave 2	Wave 3	Wave 4	Wave 5	Wave 6	Wave 7	Waves 1–7
	April 2018 to December 2019	January to May 2020	June to October 2020	November 2020 to February 2021	March to June 2021	July to December 2021	January to June 2022	July to October 2022	January 2020 to October 2022
Proportion of Cesarean sections, *n* (%)^a^	317 241/1 564 912 (20.27)	71 592/341 327 (20.97)	74 526/362 650 (20.55)	52 074/251 732 (20.69)	58 089/271 630 (21.39)	90 117/425 058 (21.20)	81 299/367 232 (22.14)	58 475/274 859 (21.27)	486 172/2 294 488 (21.19)
Proportion of COVID‐19‐positive Cesarean sections, *n* (%)^b^	0/317 241 (0.00)	21/341 327 (0.01)	395/362 650 (0.11)	619/251 732 (0.25)	1291/271 630 (0.48)	2593/425 058 (0.61)	4018/367 232 (1.09)	3454/274 859 (1.26)	12 391/2 294 488 (0.54)
Proportion of COVID‐19‐positive Cesarean sections (excluding suspicious flag), *n* (%)^b^	0/317 241 (0.00)	1–9/341 327 (0.00–0.00)	10–20/362 650 (0.00–0.01)^c^	44/251 732 (0.02)	64/271 630 (0.02)	268/425 058 (0.06)	747/367 232 (0.20)	597/274 859 (0.22)	2361/2 294 488 (0.10)
Proportion of emergency among Cesarean sections, *n* (%)^d^	128 199/317 241 (40.41)	29 443/71 592 (41.13)	30 137/74 526 (40.43)	21 531/52 074 (41.34)	24 020/58 089 (41.35)	36 871/90 117 (40.91)	34 376/81 299 (42.28)	24 183/58 475 (41.36)	200 561/486 172 (41.25)
Proportion of COVID‐19‐positive emergency among Cesarean sections, *n* (%)^e^	0/317 241 (0.00)	11/71 592 (0.02)	170/74 526 (0.23)	364/52 074 (0.70)	676/58 089 (1.16)	1307/90 117 (1.45)	2144/81 299 (2.64)	1814/58 475 (3.10)	6486/486 172 (1.33)
Proportion of COVID‐19‐positive emergency among Cesarean sections (excluding suspicious flag), *n* (%)^e^	0/317 241 (0.00)	1–9/71 592 (0.00–0.01)	10–20/74 526 (0.01–0.03)^c^	44/52 074 (0.08)	64/58 089 (0.11)	268/90 117 (0.30)	747/81 299 (0.92)	597/58 475 (1.02)	1736/486 172 (0.36)
COVID‐19 incidence (per 100 000 person‐month)^f^	0.00	2.71	13.57	67.01	74.47	126.12	996.75	2657.49	529.84

*Note*: According to the rules for using the National Database of Health Insurance Claims and Specific Health Checkup of Japan, values ranging from 1 to 9 cannot be disclosed.

Abbreviation: COVID‐19, coronavirus disease 2019.

^a^ Proportion of Cesarean sections = number of Cesarean sections/number of live births.

^b^ Proportion of COVID‐19‐positive Cesarean sections = number of COVID‐19‐positive Cesarean sections/number of live births. Calculations were made for cases including and excluding the suspicious flags, respectively.

^c^ To ensure that the number in the cell containing a value from 1 to 9 in the same row cannot be calculated from the total, this number is presented as a range rather than the actual number.

^d^ Proportion of emergency among Cesarean sections = number of emergency Cesarean sections/number of Cesarean sections.

^e^ Proportion of COVID‐19‐positive emergency among Cesarean sections = number of emergency Cesarean sections/number of Cesarean sections. Calculations were made for cases both including and excluding the suspicious flag.

^f^ COVID‐19 incidence (per 100 000 person‐months) is calculated by averaging the number of COVID‐19‐positive cases of the month divided by the Japanese population of the month.

The Poisson regression analysis with robust standard errors also revealed a statistically significant increase in the nationwide proportion of Cesarean sections during the pandemic period compared with the pre‐pandemic period (estimated risk ratio 1.042; 95% confidence interval 1.028–1.056; *p* < 0.001).

Table 3 shows the breakdown of the proportion of emergency Cesarean sections by COVID‐19 disease flag during the COVID‐19 pandemic (first to seventh waves). Among Cesarean sections with a COVID‐19 disease flag (including the suspicious flag), the proportion of emergencies was 52.34% (6486/12 391), higher than the 40.96% (200 561/486 172) in Cesarean sections without a COVID‐19 disease flag. When Cesarean sections with COVID‐19 disease flags were analyzed by the presence or absence of suspicious flags, a higher proportion of emergencies was observed in those without suspicious flags, at 73.53% (1736/2361) compared to 47.36% (4750/10 030) in those with a suspicious flag.

**TABLE 3:** Breakdown of the proportion of emergency Cesarean sections by COVID‐19 disease flag during the COVID‐19 pandemic (first to seventh waves).

	Emergency, *n* (%)	Nonemergency, *n* (%)	Total, *n*
With COVID‐19 disease flag (including suspicious flags)	6486 (52.34)	5905 (47.66)	12 391
Without a suspicious flag	1736 (73.53)	625 (26.47)	2361
With a suspicious flag	4750 (47.36)	5280 (52.64)	10 030
Without a COVID‐19 disease flag	194 075 (40.96)	279 706 (59.04)	473 781
Total	200 561 (41.25)	285 611 (58.75)	486 172

*Note*: Proportions were calculated by dividing each number by the total for the corresponding.

Abbreviation: COVID‐19, coronavirus disease 2019.

### Descriptive characteristics for each prefecture

**FIGURE 3:**
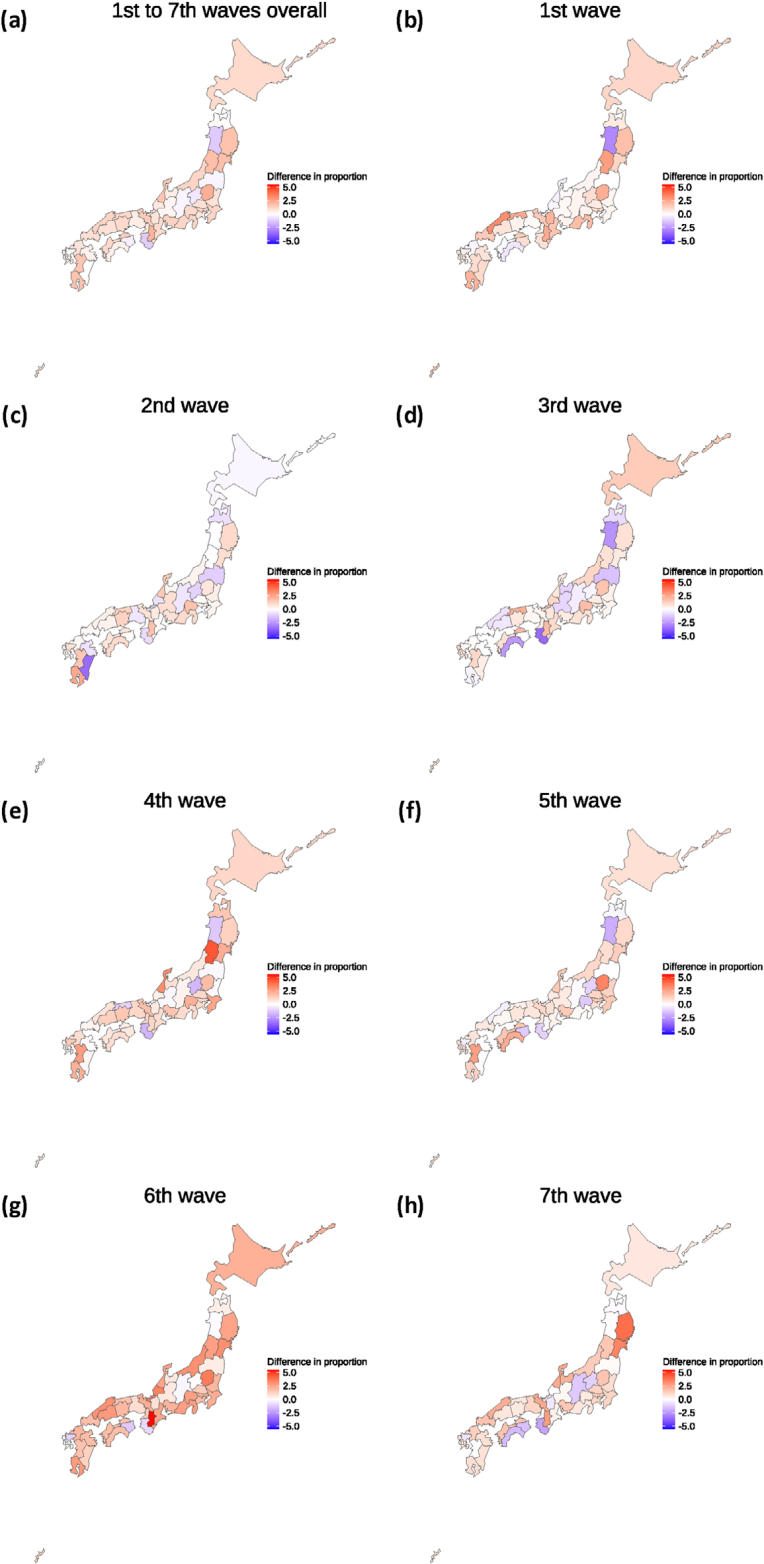
Differences in the proportion (%) of Cesarean section by prefecture before and during the COVID‐19 pandemic. (a) Differences in proportion between before and during the COVID‐19 pandemic (first to seventh waves). (b–h) Differences in proportion between before the pre‐COVID‐19 and each wave. Red indicates an increase in the proportion of Cesarean sections compared to the pre‐COVID‐19 pandemic, while blue indicates a decrease.

Figure 3 and File S3 present the descriptive characteristics of each prefecture. The differences in the proportion of Cesarean sections by prefecture between before and during the COVID‐19 pandemic are illustrated in Figure 2a. The proportion of Cesarean sections increased nationwide as well as in urban areas. Forty‐one of the 47 prefectures (87.2%) experienced a difference greater than 0. The highest difference in proportion was observed in Tochigi (2.27%), followed by Nara (2.14%), Yamagata (1.84%), Miyagi (1.84%), and Iwate (1.75%). The differences in proportion by prefecture for each wave are shown in Figure 2b–h. The most notable increase in the proportion of Cesarean sections occurred during the sixth wave, followed by a smaller increase in the seventh wave, except for some rural prefectures. Additionally, the proportion of COVID‐19‐positive Cesarean sections for each prefecture is shown in File S4.

The proportion of Cesarean sections also varied widely: in the pre‐COVID‐19 period, the highest proportion was in Kochi (2160/7687 [28.10%]), while the lowest was in Akita (1221/8529 [14.32%]). In the COVID‐19 period, the highest proportion was in Tochigi (9221/32 068 [28.75%]), and the lowest was again in Akita (1592/12 171 [13.08%]).

### Factors that could be associated with differences in the proportion of Cesarean section

The COVID‐19 incidence by prefecture was not associated with the differences in the proportion of Cesarean sections by prefecture (correlation coefficient 0.084, *p* = 0.575). The population density of inhabitable land area by prefecture, the number of obstetricians and gynecologists per 100 000 women aged 15–49 years, and the number of births per obstetrician/gynecologist were also not associated with the differences in proportion by prefecture (correlation coefficient 0.093, *p* = 0.536; correlation coefficient − 0.236, *p* = 0.110; and correlation coefficient 0.182, *p* = 0.182, respectively). These scatter plots and the values of each indicator by prefecture are shown in Files S5 and S6.

## DISCUSSION

The proportion of Cesarean sections in Japan increased 0.92% points from 20.27% before the COVID‐19 pandemic to 21.19% during the pandemic. Among live births during the pandemic, COVID‐19‐positive Cesarean sections accounted for 0.54% and 0.08%, including and excluding suspicious flags, respectively. A similar increase was observed in a model adjusting for seasonal variation. The proportion of Cesarean sections increased in 41 out of 47 prefectures, with the highest proportion observed during the sixth wave at 22.14%.

To our knowledge, this is the first study to clarify the changes in the proportion of Cesarean sections following the COVID‐19 pandemic in Japan. The results of this study are consistent with previous studies showing that the proportion of Cesarean sections increased during the COVID‐19 pandemic.^9^, ^27^ According to a systematic review and meta‐analysis of 435 studies primarily from the United States (32.4%), China (8.5%), and Spain (6.9%), the odds of Cesarean section were higher in pregnant women with COVID‐19 than in those without COVID‐19 (odds ratio 1.17, 95% confidence interval 1.01–1.36, number of studies 53).^28^

The outbreak of COVID‐19 had various effects on medical care. An analysis using secondary data from the Japan Society of Obstetrics and Gynecology, covering the years 2016 to 2020, reported a deterioration in pregnancy complications and delivery outcomes in Japan.^11^ The study suggested that this deterioration was not solely due to the direct impact of COVID‐19 infections but was also likely influenced by a combination of factors, including restrictions on antenatal checkups and increased anxiety among pregnant women during the pandemic.^29^, ^30^ Guidelines played a role in supporting daily clinical practice amid confusion. Although the proportion of Cesarean sections has increased during the COVID‐19 pandemic in Japan, it remains unclear whether Cesarean section is necessary for infection control. Now that the pandemic has settled, it is essential to reevaluate medical practices under pandemic guidelines. This study provides basic information for this purpose. Such discussions will contribute to developing appropriate responses to potential future pandemics.

Our study also revealed that the proportion of emergencies among Cesarean sections has increased by 0.81%, from 40.41% to 41.25%. During the COVID‐19 period, high proportions of emergency were observed in the COVID‐19‐positive Cesarean sections, both with (47.46%) and without suspicious flags (73.53%), compared to those without COVID‐19 a disease flag (40.96%). Although this study cannot identify the reasons for the Cesarean sections, it suggests that the emergency Cesarean section may have been performed not only on pregnant women who were reliably diagnosed with COVID‐19 but also on those suspected of having COVID‐19. Unplanned Cesarean sections can significantly impact a person's life. In Japan, there has not been sufficient discussion about the effectiveness of Cesarean sections for infection control, and this study will catalyze that discussion. A more direct survey would also be necessary to investigate the number of hospitals in Japan that opted for Cesarean sections due to COVID‐19.

Although individual‐level factors could not be thoroughly examined due to limitations in the available data, this study suggested the presence of regional variations in the proportions of Cesarean sections. The proportion of Cesarean sections before the COVID‐19 pandemic was the highest in Kochi (28.10%), followed by Miyazaki (26.84%), Tochigi (26.48%), Kagoshima (26.36%), and Gifu (24.85%); the overall proportion of Cesarean sections was 20.27% before the COVID‐19 pandemic. Akita had the lowest proportion of Cesarean sections at 14.32%, followed by Okayama (16.98%), Kanagawa (17.75%), Yamagata (18.02%), and Osaka (18.11%). A Japanese study using 2013 data similarly reported regional differences in proportions of Cesarean sections, ranging from 14.0% to 25.6% by prefecture (18.5% overall).^26^ Compared with the results of this study, the proportion of Cesarean sections in Japan had increased approximately 1.1‐fold in about 10 years.

We also investigated potential factors associated with the differences in the proportion of Cesarean sections before and during the COVID‐19 pandemic by prefecture. However, the COVID‐19 incidence by prefecture, the population density of inhabitable land area by prefecture, the number of obstetricians and gynecologists per 100 000 women aged 15–49 years, and the number of births per obstetrician/gynecologist were not associated with the differences in proportions by prefecture. It is possible that the prefectural‐level information lacked sufficient granularity in its analysis. Future studies will need more detailed data such as the municipal‐level information, which were not available in this study. Furthermore, the factors contributing to the increased difference in proportion may be complex, and the range of data available for this study did not allow us to fully examine the factors associated with the rising proportion of Cesarean sections. More detailed data that could account for this complexity would need to be collected in future studies. However, even with datasets of finer granularity or those including a wider range of factors, the possibility of unmeasured confounders remains, and results should be interpreted with caution.

Our study has some limitations. First, because of the possibility of twins and multiples, the number of deliveries could have been used as the denominator in calculating the proportion of Cesarean sections. However, data on the number of deliveries in Japan were not available; therefore, the number of births was used as the denominator. Second, the information on the disease names in the data we used was limited to COVID‐19. As a result, we were unable to evaluate the indications for Cesarean section, making it unclear whether they were performed due to COVID‐19 or not. Third, we were unable to obtain information on the type of virus, severity of infection, or vaccination status related to COVID‐19 infection in our study. Therefore, we could not take these factors into account. Further research is needed because these factors may affect the decision to perform a Cesarean section. Fourth, although this study found a slight increase in the proportion of Cesarean sections, the proportion of Cesarean sections had already been gradually increasing in Japan even before the pandemic, making it difficult to distinguish the specific impact of COVID‐19 on this increase. Owing to limitations in the available data, this study could not address this issue; however, future studies clarifying trends in the proportion of Cesarean sections following the COVID‐19 pandemic would be valuable (for example, whether the proportion declined after the pandemic subsided). Finally, we calculated the percentage of medical claims with a COVID‐19 infection indication in the same month as the Cesarean section; however, because the COVID‐19 infection may have developed later than the Cesarean section, the Cesarean sections with a COVID‐19 infection indication used in our study may have been overestimated.

In conclusion, during the COVID‐19 pandemic, the proportion of Cesarean section in Japan was increased by 0.92% compared with the period before the pandemic. Infection control measures may have contributed to this increase. Verification and preparation are warranted to respond to future pandemics.

## AUTHOR CONTRIBUTIONS

**Kensuke Shimada:** Conceptualization; data curation; formal analysis; investigation; methodology; software; visualization; writing – original draft; writing – review and editing. **Jun Komiyama:** Data curation; investigation; methodology; project administration; software; supervision; validation; visualization; writing – review and editing. **Takehiro Sugiyama:** Conceptualization; methodology; project administration; supervision; visualization; writing – review and editing. **Shin Jung‐Ho:** Data curation; investigation; software; writing – review and editing. **Tomomi Kihara:** Project administration; writing – review and editing. **Rie Masuda:** Methodology; writing – review and editing. **Susumu Kunisawa:** Project administration; writing – review and editing. **Masao Iwagami:** Methodology; supervision; writing – review and editing. **Isao Muraki:** Validation; writing – review and editing. **Yuichi Imanaka:** Funding acquisition; project administration; resources; writing – review and editing. **Hiroyasu Iso:** Funding acquisition; project administration; supervision; writing – review and editing. **Nanako Tamiya:** Conceptualization; funding acquisition; methodology; project administration; resources; supervision; writing – review and editing.

## CONFLICT OF INTEREST STATEMENT

The authors declare no conflict of interests for this article.

## Supporting information


**File S1.** Proportion of Cesarean sections and the proportion of the emergency among Cesarean sections.

**File S2.** Number of patients with COVID‐19 in Japan.

**File S3.** Proportion of Cesarean sections in each prefecture during the COVID‐19 pandemic.

**File S4.** Proportion of COVID‐19‐positive Cesarean sections (including suspicious flag) in each prefecture during the COVID‐19 pandemic.

**File S5.** Scatter plots of COVID‐19 incidence, population density of inhabitable land area, the number of obstetricians and gynecologists per 100,000 women aged 15–49 years, and the number of births per obstetrician/gynecologist compared to the difference in the proportion of Cesarean sections by prefecture.

**File S6.** Difference in the proportion of Cesarean section, COVID‐19 incidence, the population density of inhabitable land area, the number of obstetricians and gynecologists per 100,000 women aged 15–49 years, and the number of births per obstetrician/gynecologist by prefecture.

## Data Availability

The research data are not allowed to be shared because of the relevant laws and guidelines in Japan.
