# Supplementary material for: Changes in proportions of Cesarean section before and during the COVID‐19 pandemic in Japan
Source: J Obstet Gynaecol Res. 2025 Jul 10;51(7):e16370. doi: 10.1111/jog.16370 (PMC12242368; doi:10.1111/jog.16370)
Supplement: Supplementary file 1 — File S1. Proportion of Cesarean sections and the proportion of the emergency among Cesarean sections. [file JOG-51-0-s001.docx]

**File S1.** Proportion of Cesarean sections and the proportion of the emergency among Cesarean sections

| **Year/Month** | **Birth, n** | **Cesarean section, n (%)^a^** | **COVID-19-positive Cesarean sections, n (%)^b^** | **COVID-19-positive Cesarean sections (excluding suspicious flags), n (%)^b^** | **Emergency Cesarean sections, n (%)^c^** | **COVID-19-positive emergency Cesarean sections, n (%)^d^** | **COVID-19-positive emergency Cesarean sections (excluding suspicious flag), n (%)^d^** |
| --- | --- | --- | --- | --- | --- | --- | --- |
| 2018/04 | 73,873 | 14,915 (20.19) | 0 (0.00) | 0 (0.00) | 5,941 (39.83) | 0 (0.00) | 0 (0.00) |
| 2018/05 | 79,745 | 16,194 (20.31) | 0 (0.00) | 0 (0.00) | 6,399 (39.51) | 0 (0.00) | 0 (0.00) |
| 2018/06 | 75,628 | 15,473 (20.46) | 0 (0.00) | 0 (0.00) | 6,104 (39.45) | 0 (0.00) | 0 (0.00) |
| 2018/07 | 80,022 | 16,055 (20.06) | 0 (0.00) | 0 (0.00) | 6,431 (40.06) | 0 (0.00) | 0 (0.00) |
| 2018/08 | 80,705 | 16,321 (20.22) | 0 (0.00) | 0 (0.00) | 6,472 (39.65) | 0 (0.00) | 0 (0.00) |
| 2018/09 | 76,697 | 14,781 (19.27) | 0 (0.00) | 0 (0.00) | 5,939 (40.18) | 0 (0.00) | 0 (0.00) |
| 2018/10 | 79,792 | 15,909 (19.94) | 0 (0.00) | 0 (0.00) | 6,384 (40.13) | 0 (0.00) | 0 (0.00) |
| 2018/11 | 74,773 | 15,105 (20.20) | 0 (0.00) | 0 (0.00) | 6,152 (40.73) | 0 (0.00) | 0 (0.00) |
| 2018/12 | 78,470 | 15,254 (19.44) | 0 (0.00) | 0 (0.00) | 6,077 (39.84) | 0 (0.00) | 0 (0.00) |
| 2019/01 | 69,709 | 14,578 (20.91) | 0 (0.00) | 0 (0.00) | 6,016 (41.27) | 0 (0.00) | 0 (0.00) |
| 2019/02 | 64,340 | 13,444 (20.90) | 0 (0.00) | 0 (0.00) | 5,346 (39.76) | 0 (0.00) | 0 (0.00) |
| 2019/03 | 68,967 | 13,822 (20.04) | 0 (0.00) | 0 (0.00) | 5,808 (42.02) | 0 (0.00) | 0 (0.00) |
| 2019/04 | 68,802 | 14,837 (21.57) | 0 (0.00) | 0 (0.00) | 6,017 (40.55) | 0 (0.00) | 0 (0.00) |
| 2019/05 | 76,583 | 15,185 (19.83) | 0 (0.00) | 0 (0.00) | 6,188 (40.75) | 0 (0.00) | 0 (0.00) |
| 2019/06 | 70,699 | 14,357 (20.31) | 0 (0.00) | 0 (0.00) | 6,023 (41.95) | 0 (0.00) | 0 (0.00) |
| 2019/07 | 76,237 | 15,716 (20.62) | 0 (0.00) | 0 (0.00) | 6,282 (39.97) | 0 (0.00) | 0 (0.00) |
| 2019/08 | 75,730 | 15,646 (20.66) | 0 (0.00) | 0 (0.00) | 6,382 (40.79) | 0 (0.00) | 0 (0.00) |
| 2019/09 | 73,990 | 14,336 (19.38) | 0 (0.00) | 0 (0.00) | 5,907 (41.20) | 0 (0.00) | 0 (0.00) |
| 2019/10 | 74,472 | 15,322 (20.57) | 0 (0.00) | 0 (0.00) | 6,128 (39.99) | 0 (0.00) | 0 (0.00) |
| 2019/11 | 69,863 | 14,402 (20.62) | 0 (0.00) | 0 (0.00) | 6,038 (41.92) | 0 (0.00) | 0 (0.00) |
| 2019/12 | 75,815 | 15,589 (20.56) | 0 (0.00) | 0 (0.00) | 6,165 (39.55) | 0 (0.00) | 0 (0.00) |
| 2020/01 | 68,147 | 14,377 (21.10) | 0 (0.00) | 0 (0.00) | 6,010 (41.80) | 0 (0.00) | 0 (0.00) |
| 2020/02 | 64,037 | 13,576 (21.20) | 0 (0.00) | 0 (0.00) | 5,516 (40.63) | 0 (0.00) | 0 (0.00) |
| 2020/03 | 68,511 | 14,144 (20.65) | 0 (0.00) | 0 (0.00) | 5,909 (41.78) | 0 (0.00) | 0 (0.00) |
| 2020/04 | 71,097 | 15,344 (21.58) | 1–9 (0.00–0.01)^e^ | 1–9 (0.00–0.01)^e^ | 6,114 (39.85) | 1–9 (0.01–0.06)^e^ | 1–9 (0.01–0.06)^e^ |
| 2020/05 | 69,535 | 14,151 (20.35) | 10–20 (0.01–0.03)^f^ | 1–9 (0.00–0.01)^e^ | 5,894 (41.65) | 1–9 (0.01–0.06)^e^ | 1–9 (0.01–0.06)^e^ |
| 2020/06 | 70,447 | 14,735 (20.92) | 39 (0.06) | 1–9 (0.00–0.01)^e^ | 5,984 (40.61) | 13 (0.09) | 0 (0.00) |
| 2020/07 | 74,217 | 15,682 (21.13) | 69 (0.09) | 1–9 (0.00–0.01)^e^ | 6,315 (40.27) | 31 (0.10) | 1–9 (0.01–0.06)^e^ |
| 2020/08 | 72,983 | 14,438 (19.78) | 90 (0.12) | 1–9 (0.00–0.01)^e^ | 5,970 (41.35) | 40 (0.28) | 1–9 (0.01–0.06)^e^ |
| 2020/09 | 73,048 | 14,705 (20.13) | 82 (0.11) | 1–9 (0.00–0.01)^e^ | 5,852 (39.80) | 37 (0.25) | 1–9 (0.01–0.06)^e^ |
| 2020/10 | 71,955 | 14,966 (20.80) | 115 (0.16) | 1–9 (0.00–0.01)^e^ | 6,016 (40.20) | 49 (0.33) | 1–9 (0.01–0.06)^e^ |
| 2020/11 | 66,719 | 13,495 (20.23) | 112 (0.17) | 1–9 (0.00–0.01)^e^ | 5,597 (41.47) | 58 (0.43) | 1–9 (0.01–0.07)^e^ |
| 2020/12 | 70,108 | 13,869 (19.78) | 165 (0.24) | 16 (0.02) | 5,720 (41.24) | 98 (0.71) | 12 (0.09) |
| 2021/01 | 57,573 | 12,426 (21.58) | 175 (0.30) | 27 (0.05) | 5,251 (42.26) | 116 (0.93) | 23 (0.19) |
| 2021/02 | 57,332 | 12,284 (21.43) | 167 (0.29) | 10–20 (0.02–0.03)^f^ | 4,963 (40.40) | 92 (0.75) | 1–9 (0.01–0.07)^e^ |
| 2021/03 | 66,702 | 14,348 (21.51) | 229 (0.34) | 1–9 (0.00–0.01)^e^ | 5,928 (41.32) | 131 (0.91) | 1–9 (0.01–0.06)^e^ |
| 2021/04 | 68,270 | 15,521 (22.74) | 320 (0.47) | 31 (0.05) | 6,246 (40.24) | 166 (1.07) | 23 (0.15) |
| 2021/05 | 68,111 | 13,623 (20.00) | 344 (0.51) | 35 (0.05) | 5,887 (43.21) | 187 (1.37) | 26 (0.19) |
| 2021/06 | 68,547 | 14,597 (21.30) | 398 (0.58) | 10–20 (0.01–0.03)^f^ | 5,959 (40.82) | 192 (1.32) | 10–20 (0.07–0.14)^f^ |
| 2021/07 | 71,726 | 15,495 (21.60) | 374 (0.52) | 37 (0.05) | 6,312 (40.74) | 162 (1.05) | 32 (0.21) |
| 2021/08 | 72,998 | 15,193 (20.81) | 575 (0.79) | 173 (0.24) | 6,432 (42.34) | 328 (2.16) | 139 (0.91) |
| 2021/09 | 72,757 | 15,356 (21.17) | 502 (0.69) | 100 (0.14) | 6,191 (40.32) | 275 (1.79) | 80 (0.52) |
| 2021/10 | 70,298 | 14,839 (21.11) | 396 (0.56) | 16 (0.02) | 6,095 (41.07) | 181 (1.22) | 1–9 (0.01–0.06)^e^ |
| 2021/11 | 67,603 | 14,420 (21.33) | 363 (0.54) | 1–9 (0.00–0.01)^e^ | 5,853 (40.59) | 165 (1.14) | 1–9 (0.01–0.06)^e^ |
| 2021/12 | 69,676 | 14,814 (21.26) | 383 (0.55) | 1–9 (0.00–0.01)^e^ | 5,988 (40.42) | 196 (1.32) | 1–9 (0.01–0.06)^e^ |
| 2022/01 | 61,486 | 13,740 (22.35) | 600 (0.98) | 151 (0.25) | 5,881 (42.80) | 308 (2.24) | 105 (0.76) |
| 2022/02 | 56,958 | 12,501 (21.95) | 762 (1.34) | 275 (0.48) | 5,194 (41.55) | 428 (3.42) | 205 (1.64) |
| 2022/03 | 62,714 | 13,695 (21.84) | 673 (1.07) | 196 (0.31) | 5,828 (42.56) | 374 (2.73) | 151 (1.10) |
| 2022/04 | 60,924 | 14,006 (22.99) | 758 (1.24) | 190 (0.31) | 5,857 (41.82) | 390 (2.78) | 139 (0.99) |
| 2022/05 | 62,866 | 13,312 (21.18) | 625 (0.99) | 130 (0.21) | 5,810 (43.64) | 326 (2.45) | 98 (0.74) |
| 2022/06 | 62,284 | 14,045 (22.55) | 600 (0.96) | 71 (0.11) | 5,806 (41.34) | 318 (2.26) | 49 (0.35) |
| 2022/07 | 65,885 | 14,764 (22.41) | 890 (1.35) | 280 (0.42) | 6,193 (41.95) | 498 (3.37) | 217 (1.47) |
| 2022/08 | 71,363 | 15,513 (21.74) | 1,107 (1.55) | 364 (0.51) | 6,462 (41.66) | 615 (3.96) | 258 (1.66) |
| 2022/09 | 70,032 | 14,873 (21.24) | 813 (1.16) | 145 (0.21) | 5,964 (40.10) | 401 (2.70) | 97 (0.65) |
| 2022/10 | 67,579 | 13,325 (19.72) | 644 (0.95) | 44 (0.07) | 5,564 (41.76) | 300 (2.25) | 25 (0.19) |

COVID-19: coronavirus disease 2019.

^a^Proportion of Cesarean sections = number of Cesarean sections / number of live births.

^b^Proportion of COVID-19-positive Cesarean sections = number of COVID-19-positive Cesarean sections / number of live births. Calculations were made for cases including and excluding the suspicious flags, respectively.

^c^Proportion of emergency among Cesarean sections = number of emergency Cesarean sections / number of Cesarean sections.

^d^Proportion of COVID-19-positive emergency among Cesarean sections = number of COVID-19-positive emergency Cesarean sections / number of Cesarean sections. Calculations were made for cases including and excluding the suspicious flags, respectively.

^e^Items with a total number between 1 and 9 are not allowed to be published; therefore, they are listed in a range (1–9), and the percentages are provided as a range accordingly.

^f^To ensure that the number in the cell with a value between 1 to 9 in the same row cannot be derived from the total, this number is shown as a range rather than the actual number.
