# Supplementary material for: Changes in proportions of Cesarean section before and during the COVID‐19 pandemic in Japan
Source: J Obstet Gynaecol Res. 2025 Jul 10;51(7):e16370. doi: 10.1111/jog.16370 (PMC12242368; doi:10.1111/jog.16370)
Supplement: Supplementary file 2 — File S2. Number of patients with COVID‐19 in Japan. [file JOG-51-0-s003.docx]

**File S2.** Number of patients with COVID-19 in Japan

| **Year/Month** | **Number of COVID-19 positive, n** | **Japanese population, n** | **COVID-19 incidence (per 100,000 person-month)^a^** |
| --- | --- | --- | --- |
| 2018/04 | 0 | 124,413,358 | 0.00 |
| 2018/05 | 0 | 124,354,431 | 0.00 |
| 2018/06 | 0 | 124,331,066 | 0.00 |
| 2018/07 | 0 | 124,349,004 | 0.00 |
| 2018/08 | 0 | 124,352,776 | 0.00 |
| 2018/09 | 0 | 124,259,024 | 0.00 |
| 2018/10 | 0 | 124,218,285 | 0.00 |
| 2018/11 | 0 | 124,181,867 | 0.00 |
| 2018/12 | 0 | 124,144,438 | 0.00 |
| 2019/01 | 0 | 124,193,600 | 0.00 |
| 2019/02 | 0 | 124,057,626 | 0.00 |
| 2019/03 | 0 | 123,992,541 | 0.00 |
| 2019/04 | 0 | 123,960,421 | 0.00 |
| 2019/05 | 0 | 123,900,068 | 0.00 |
| 2019/06 | 0 | 123,873,418 | 0.00 |
| 2019/07 | 0 | 123,881,011 | 0.00 |
| 2019/08 | 0 | 123,880,151 | 0.00 |
| 2019/09 | 0 | 123,774,676 | 0.00 |
| 2019/10 | 0 | 123,731,176 | 0.00 |
| 2019/11 | 0 | 123,688,713 | 0.00 |
| 2019/12 | 0 | 123,645,532 | 0.00 |
| 2020/01 | 3 | 123,688,084 | 0.00 |
| 2020/02 | 69 | 123,549,942 | 0.06 |
| 2020/03 | 489 | 123,458,318 | 0.40 |
| 2020/04 | 3,747 | 123,423,966 | 3.04 |
| 2020/05 | 958 | 123,400,940 | 0.78 |
| 2020/06 | 994 | 123,369,649 | 0.81 |
| 2020/07 | 6,464 | 123,352,466 | 5.24 |
| 2020/08 | 8,125 | 123,334,135 | 6.59 |
| 2020/09 | 4,918 | 123,288,012 | 3.99 |
| 2020/10 | 5,350 | 123,250,274 | 4.34 |
| 2020/11 | 9,861 | 123,204,724 | 8.00 |
| 2020/12 | 19,369 | 123,155,341 | 15.73 |
| 2021/01 | 40,367 | 123,260,632 | 32.75 |
| 2021/02 | 10,997 | 123,160,154 | 8.93 |
| 2021/03 | 9,310 | 123,097,165 | 7.56 |
| 2021/04 | 28,992 | 123,049,873 | 23.56 |
| 2021/05 | 21,871 | 122,997,629 | 17.78 |
| 2021/06 | 12,977 | 122,952,071 | 10.55 |
| 2021/07 | 44,448 | 122,928,206 | 36.16 |
| 2021/08 | 129,193 | 122,898,123 | 105.12 |
| 2021/09 | 31,196 | 122,834,275 | 25.40 |
| 2021/10 | 2,892 | 122,780,487 | 2.36 |
| 2021/11 | 665 | 122,728,075 | 0.54 |
| 2021/12 | 945 | 122,674,226 | 0.77 |
| 2022/01 | 207,692 | 122,638,231 | 169.35 |
| 2022/02 | 394,030 | 122,529,576 | 321.58 |
| 2022/03 | 247,810 | 122,443,720 | 202.39 |
| 2022/04 | 184,860 | 122,387,920 | 151.04 |
| 2022/05 | 100,826 | 122,325,387 | 82.42 |
| 2022/06 | 58,999 | 122,272,008 | 48.25 |
| 2022/07 | 567,960 | 122,262,962 | 464.54 |
| 2022/08 | 759,118 | 122,237,965 | 621.02 |
| 2022/09 | 243,973 | 122,104,154 | 199.81 |
| 2022/10 | 100,143 | 122,030,523 | 82.06 |

COVID-19: coronavirus disease 2019.

^a^COVID-19 incidence = number of COVID-19-positive cases per month in Japan / Japanese population on the corresponding month.
