# Supplementary material for: Changes in proportions of Cesarean section before and during the COVID‐19 pandemic in Japan
Source: J Obstet Gynaecol Res. 2025 Jul 10;51(7):e16370. doi: 10.1111/jog.16370 (PMC12242368; doi:10.1111/jog.16370)
Supplement: Supplementary file 3 — File S3. Proportion of Cesarean sections in each prefecture during the COVID‐19 pandemic. [file JOG-51-0-s002.docx]

**File S3.** Proportion of Cesarean sections in each prefecture during the COVID-19 pandemic

| **Prefecture** | **Number of Cesarean sections / number of births (proportion of Cesarean sections^a^, %)** | | | | | | | | |
| --- | --- | --- | --- | --- | --- | --- | --- | --- | --- |
|  | **Pre-COVID-19 period** | **During COVID-19 period** | | | | | | | |
|  |  | **Wave 1** | **Wave 2** | **Wave 3** | **Wave 4** | **Wave 5** | **Wave 6** | **Wave 7** | **Wave 1 to 7** |
|  | April 2018 to December 2019 | January to May 2020 | June to October 2020 | November 2020 to February 2021 | March to June 2021 | July to December 2021 | January to June 2022 | July to October 2022 | January 2020 to October 2022 |
| Hokkaido | 12,298 / 55,830 (22.03) | 2,829 / 12,205 (23.18) | 2,749 / 12,605 (21.81) | 2,078 / 8,841 (23.50) | 2,271 / 9,789 (23.20) | 3,401 / 14,844 (22.91) | 3,126 / 12,885 (24.26) | 2,100 / 9,246 (22.71) | 18,554 / 80,415 (23.07) |
| Aomori | 2,551 / 13,034 (19.57) | 573 / 2,839 (20.18) | 558 / 2,964 (18.83) | 372 / 1,986 (18.73) | 476 / 2,247 (21.18) | 642 / 3,314 (19.37) | 593 / 2,954 (20.07) | 401 / 2,058 (19.48) | 3,615 / 18,362 (19.69) |
| Iwate | 3,070 / 12,794 (24.00) | 712 / 2,760 (25.80) | 727 / 2,898 (25.09) | 501 / 2,015 (24.86) | 543 / 2,145 (25.31) | 846 / 3,372 (25.09) | 749 / 2,819 (26.57) | 560 / 2,003 (27.96) | 4,638 / 18,012 (25.75) |
| Miyagi | 5,827 / 27,307 (21.34) | 1,336 / 5,914 (22.59) | 1,390 / 6,235 (22.29) | 949 / 4,324 (21.95) | 1,108 / 4,681 (23.67) | 1,613 / 7,087 (22.76) | 1,533 / 6,265 (24.47) | 1,113 / 4,499 (24.74) | 9,042 / 39,005 (23.18) |
| Akita | 1,221 / 8,529 (14.32) | 210 / 1,826 (11.50) | 278 / 1,949 (14.26) | 159 / 1,356 (11.73) | 196 / 1,505 (13.02) | 272 / 2,198 (12.37) | 282 / 1,983 (14.22) | 195 / 1,354 (14.40) | 1,592 / 12,171 (13.08) |
| Yamagata | 2,113 / 11,726 (18.02) | 532 / 2,562 (20.77) | 486 / 2,689 (18.07) | 336 / 1,779 (18.89) | 445 / 1,976 (22.52) | 590 / 3,109 (18.98) | 551 / 2,661 (20.71) | 404 / 2,058 (19.63) | 3,344 / 16,834 (19.86) |
| Fukushima | 5,084 / 21,197 (23.98) | 1,068 / 4,451 (23.99) | 1,116 / 4,881 (22.86) | 771 / 3,422 (22.53) | 853 / 3,583 (23.81) | 1,325 / 5,527 (23.97) | 1,145 / 4,666 (24.54) | 823 / 3,410 (24.13) | 7,101 / 29,940 (23.72) |
| Ibaraki | 6,669 / 32,533 (20.50) | 1,502 / 7,237 (20.75) | 1,534 / 7,311 (20.98) | 1,087 / 5,197 (20.92) | 1,146 / 5,623 (20.38) | 1,856 / 8,523 (21.78) | 1,735 / 7,685 (22.58) | 1,209 / 5,628 (21.48) | 10,069 / 47,204 (21.33) |
| Tochigi | 6,078 / 22,953 (26.48) | 1,388 / 4,824 (28.77) | 1,392 / 5,113 (27.22) | 1,006 / 3,573 (28.16) | 1,078 / 3,800 (28.37) | 1,784 / 5,971 (29.88) | 1,523 / 5,056 (30.12) | 1,050 / 3,731 (28.14) | 9,221 / 32,068 (28.75) |
| Gunma | 4,891 / 21,748 (22.49) | 1,124 / 4,791 (23.46) | 1,066 / 4,971 (21.44) | 773 / 3,475 (22.24) | 789 / 3,790 (20.82) | 1,260 / 5,869 (21.47) | 1,138 / 5,041 (22.57) | 826 / 3,861 (21.39) | 6,976 / 31,798 (21.94) |
| Saitama | 17,616 / 87,654 (20.10) | 3,983 / 19,274 (20.67) | 4,108 / 20,350 (20.19) | 2,885 / 13,984 (20.63) | 3,197 / 15,060 (21.23) | 5,129 / 24,083 (21.30) | 4,424 / 20,547 (21.53) | 3,334 / 15,577 (21.40) | 27,060 / 128,875 (21.00) |
| Chiba | 15,236 / 74,044 (20.58) | 3,410 / 16,336 (20.87) | 3,611 / 17,293 (20.88) | 2,477 / 11,861 (20.88) | 2,962 / 12,820 (23.10) | 4,489 / 20,283 (22.13) | 4,045 / 17,643 (22.93) | 2,904 / 13,305 (21.83) | 23,898 / 109,541 (21.82) |
| Tokyo | 35,151 / 184,201 (19.08) | 8,144 / 40,407 (20.15) | 8,474 / 43,407 (19.52) | 5,620 / 28,550 (19.68) | 6,446 / 32,195 (20.02) | 10,409 / 50,504 (20.61) | 9,186 / 42,917 (21.40) | 6,759 / 32,942 (20.52) | 55,038 / 270,922 (20.32) |
| Kanagawa | 20,203 / 113,795 (17.75) | 4,416 / 24,653 (17.91) | 4,620 / 26,327 (17.55) | 3,171 / 17,900 (17.72) | 3,678 / 19,579 (18.79) | 5,835 / 31,242 (18.68) | 5,240 / 26,747 (19.59) | 3,868 / 20,419 (18.94) | 30,828 / 166,867 (18.47) |
| Niigata | 4,715 / 24,529 (19.22) | 1,023 / 5,239 (19.53) | 1,110 / 5,673 (19.57) | 809 / 3,963 (20.41) | 839 / 4,216 (19.90) | 1,322 / 6,497 (20.35) | 1,276 / 5,714 (22.33) | 871 / 4,206 (20.71) | 7,250 / 35,508 (20.42) |
| Toyama | 2,174 / 11,775 (18.46) | 467 / 2,508 (18.62) | 511 / 2,730 (18.72) | 323 / 1,853 (17.43) | 399 / 2,060 (19.37) | 597 / 3,181 (18.77) | 562 / 2,940 (19.12) | 437 / 2,112 (20.69) | 3,296 / 17,384 (18.96) |
| Ishikawa | 3,118 / 14,119 (22.08) | 687 / 3,165 (21.71) | 784 / 3,316 (23.64) | 525 / 2,301 (22.82) | 602 / 2,384 (25.25) | 900 / 3,803 (23.67) | 849 / 3,442 (24.67) | 602 / 2,454 (24.53) | 4,949 / 20,865 (23.72) |
| Fukui | 2,252 / 9,776 (23.04) | 497 / 2,179 (22.81) | 504 / 2,254 (22.36) | 384 / 1,587 (24.20) | 414 / 1,765 (23.46) | 650 / 2,751 (23.63) | 592 / 2,232 (26.52) | 416 / 1,806 (23.03) | 3,457 / 14,574 (23.72) |
| Yamanashi | 1,838 / 9,400 (19.55) | 411 / 2,060 (19.95) | 486 / 2,283 (21.29) | 328 / 1,532 (21.41) | 356 / 1,632 (21.81) | 483 / 2,642 (18.28) | 515 / 2,314 (22.26) | 342 / 1,693 (20.20) | 2,921 / 14,156 (20.63) |
| Nagano | 4,703 / 24,382 (19.29) | 996 / 5,080 (19.61) | 1,047 / 5,598 (18.70) | 734 / 3,888 (18.88) | 819 / 4,173 (19.63) | 1,273 / 6,637 (19.18) | 1,091 / 5,718 (19.08) | 778 / 4,315 (18.03) | 6,738 / 35,409 (19.03) |
| Gifu | 5,787 / 23,291 (24.85) | 1,227 / 4,901 (25.04) | 1,331 / 5,162 (25.78) | 887 / 3,715 (23.88) | 951 / 3,858 (24.65) | 1,575 / 6,186 (25.46) | 1,352 / 5,297 (25.52) | 978 / 3,898 (25.09) | 8,301 / 33,017 (25.14) |
| Shizuoka | 8,898 / 42,959 (20.71) | 1,980 / 8,773 (22.57) | 2,130 / 9,790 (21.76) | 1,447 / 6,711 (21.56) | 1,576 / 7,249 (21.74) | 2,411 / 11,545 (20.88) | 2,250 / 9,592 (23.46) | 1,563 / 7,304 (21.40) | 13,357 / 60,964 (21.91) |
| Aich | 21,771 / 103,546 (21.03) | 4,814 / 22,511 (21.39) | 5,263 / 24,066 (21.87) | 3,687 / 17,087 (21.58) | 4,043 / 17,819 (22.69) | 6,318 / 28,048 (22.53) | 5,657 / 24,426 (23.16) | 4,126 / 18,229 (22.63) | 33,908 / 152,186 (22.28) |
| Mie | 4,183 / 21,351 (19.59) | 958 / 4,507 (21.26) | 941 / 4,761 (19.76) | 696 / 3,372 (20.64) | 737 / 3,581 (20.58) | 1,180 / 5,898 (20.01) | 1,110 / 5,017 (22.12) | 715 / 3,581 (19.97) | 6,337 / 30,717 (20.63) |
| Shiga | 4,033 / 19,460 (20.72) | 945 / 4,132 (22.87) | 968 / 4,537 (21.34) | 669 / 3,118 (21.46) | 714 / 3,344 (21.35) | 1,174 / 5,435 (21.60) | 962 / 4,448 (21.63) | 718 / 3,559 (20.17) | 6,150 / 28,573 (21.52) |
| Kyoto | 6,211 / 30,507 (20.36) | 1,452 / 6,639 (21.87) | 1,503 / 7,102 (21.16) | 1,025 / 4,961 (20.66) | 1,198 / 5,402 (22.18) | 1,754 / 8,154 (21.51) | 1,639 / 7,217 (22.71) | 1,229 / 5,386 (22.82) | 9,800 / 44,861 (21.85) |
| Osaka | 20,327 / 112,272 (18.11) | 4,664 / 25,226 (18.49) | 4,855 / 26,698 (18.18) | 3,483 / 18,570 (18.76) | 3,862 / 19,917 (19.39) | 5,870 / 31,246 (18.79) | 5,334 / 27,239 (19.58) | 3,775 / 20,531 (18.39) | 31,843 / 169,427 (18.79) |
| Hyogo | 13,603 / 68,340 (19.90) | 3,029 / 15,085 (20.08) | 3,079 / 15,935 (19.32) | 2,203 / 10,933 (20.15) | 2,485 / 11,796 (21.07) | 3,874 / 18,784 (20.62) | 3,372 / 16,144 (20.89) | 2,510 / 11,889 (21.11) | 20,552 / 100,566 (20.44) |
| Nara | 3,361 / 15,179 (22.14) | 777 / 3,168 (24.53) | 791 / 3,310 (23.90) | 569 / 2,367 (24.04) | 607 / 2,606 (23.29) | 908 / 4,131 (21.98) | 949 / 3,437 (27.61) | 646 / 2,593 (24.91) | 5,247 / 21,612 (24.28) |
| Wakayama | 2,255 / 10,410 (21.66) | 532 / 2,363 (22.51) | 507 / 2,434 (20.83) | 321 / 1,771 (18.13) | 373 / 1,872 (19.93) | 578 / 2,805 (20.61) | 516 / 2,480 (20.81) | 364 / 1,866 (19.51) | 3,191 / 15,591 (20.47) |
| Tottori | 1,533 / 7,166 (21.39) | 363 / 1,522 (23.85) | 370 / 1,630 (22.70) | 277 / 1,171 (23.65) | 254 / 1,250 (20.32) | 419 / 1,918 (21.85) | 413 / 1,785 (23.14) | 291 / 1,319 (22.06) | 2,387 / 10,595 (22.53) |
| Shimane | 1,764 / 8,259 (21.36) | 433 / 1,763 (24.56) | 428 / 1,963 (21.80) | 283 / 1,370 (20.66) | 323 / 1,458 (22.15) | 490 / 2,334 (20.99) | 484 / 1,982 (24.42) | 346 / 1,461 (23.68) | 2,787 / 12,331 (22.60) |
| Okayama | 4,197 / 24,722 (16.98) | 956 / 5,507 (17.36) | 1,076 / 5,895 (18.25) | 727 / 4,117 (17.66) | 807 / 4,342 (18.59) | 1,197 / 6,767 (17.69) | 1,136 / 5,973 (19.02) | 777 / 4,416 (17.60) | 6,676 / 37,017 (18.03) |
| Hiroshima | 7,021 / 36,224 (19.38) | 1,569 / 7,782 (20.16) | 1,664 / 8,404 (19.80) | 1,121 / 5,958 (18.82) | 1,310 / 6,317 (20.74) | 1,918 / 9,780 (19.61) | 1,851 / 8,314 (22.26) | 1,294 / 6,434 (20.11) | 10,727 / 52,989 (20.24) |
| Yamaguchi | 2,835 / 15,581 (18.20) | 663 / 3,361 (19.73) | 650 / 3,533 (18.40) | 451 / 2,487 (18.13) | 492 / 2,658 (18.51) | 781 / 4,141 (18.86) | 742 / 3,704 (20.03) | 556 / 2,797 (19.88) | 4,335 / 22,681 (19.11) |
| Tokushima | 1,749 / 8,323 (21.01) | 402 / 1,828 (21.99) | 415 / 1,965 (21.12) | 292 / 1,394 (20.95) | 311 / 1,436 (21.66) | 443 / 2,235 (19.82) | 390 / 1,976 (19.74) | 291 / 1,490 (19.53) | 2,544 / 12,324 (20.64) |
| Kagawa | 2,249 / 11,836 (19.00) | 518 / 2,535 (20.43) | 521 / 2,635 (19.77) | 389 / 1,852 (21.00) | 421 / 2,162 (19.47) | 664 / 3,218 (20.63) | 578 / 2,780 (20.79) | 415 / 2,006 (20.69) | 3,506 / 17,188 (20.40) |
| Ehime | 3,272 / 15,546 (21.05) | 698 / 3,339 (20.90) | 745 / 3,422 (21.77) | 555 / 2,528 (21.95) | 596 / 2,743 (21.73) | 925 / 4,081 (22.67) | 811 / 3,532 (22.96) | 618 / 2,717 (22.75) | 4,948 / 22,362 (22.13) |
| Kochi | 2,160 / 7,687 (28.10) | 475 / 1,726 (27.52) | 485 / 1,663 (29.16) | 339 / 1,321 (25.66) | 401 / 1,381 (29.04) | 634 / 2,081 (30.47) | 528 / 1,763 (29.95) | 354 / 1,335 (26.52) | 3,216 / 11,270 (28.54) |
| Fukuoka | 13,366 / 71,480 (18.70) | 2,952 / 15,970 (18.48) | 3,154 / 16,696 (18.89) | 2,217 / 11,788 (18.81) | 2,483 / 12,635 (19.65) | 3,786 / 19,417 (19.50) | 3,597 / 17,293 (20.80) | 2,557 / 12,752 (20.05) | 20,746 / 106,551 (19.47) |
| Saga | 2,189 / 11,176 (19.59) | 494 / 2,465 (20.04) | 530 / 2,591 (20.46) | 359 / 1,820 (19.73) | 415 / 1,970 (21.07) | 577 / 3,011 (19.16) | 503 / 2,705 (18.60) | 370 / 1,947 (19.00) | 3,248 / 16,509 (19.67) |
| Nagasaki | 3,269 / 17,251 (18.95) | 740 / 3,763 (19.67) | 756 / 3,918 (19.30) | 560 / 2,789 (20.08) | 602 / 3,120 (19.29) | 889 / 4,454 (19.96) | 757 / 3,983 (19.01) | 594 / 2,982 (19.92) | 4,898 / 25,009 (19.58) |
| Kumamoto | 5,510 / 24,135 (22.83) | 1,273 / 5,344 (23.82) | 1,362 / 5,581 (24.40) | 967 / 4,035 (23.97) | 1,069 / 4,180 (25.57) | 1,671 / 6,541 (25.55) | 1,422 / 5,786 (24.58) | 958 / 4,191 (22.86) | 8,722 / 35,658 (24.46) |
| Oita | 3,051 / 13,780 (22.14) | 716 / 3,119 (22.96) | 703 / 3,270 (21.50) | 508 / 2,305 (22.04) | 545 / 2,469 (22.07) | 830 / 3,746 (22.16) | 789 / 3,284 (24.03) | 556 / 2,415 (23.02) | 4,647 / 20,608 (22.55) |
| Miyazaki | 3,881 / 14,461 (26.84) | 891 / 3,195 (27.89) | 756 / 3,241 (23.33) | 661 / 2,418 (27.34) | 681 / 2,511 (27.12) | 1,055 / 3,944 (26.75) | 962 / 3,415 (28.17) | 696 / 2,514 (27.68) | 5,702 / 21,238 (26.85) |
| Kagoshima | 5,742 / 21,785 (26.36) | 1,309 / 4,591 (28.51) | 1,447 / 5,028 (28.78) | 963 / 3,692 (26.08) | 1,079 / 3,778 (28.56) | 1,703 / 6,166 (27.62) | 1,459 / 4,998 (29.19) | 996 / 3,649 (27.30) | 8,956 / 31,902 (28.07) |
| Okinawa | 6,216 / 26,859 (23.14) | 1,454 / 5,902 (24.64) | 1,545 / 6,573 (23.51) | 1,130 / 4,695 (24.07) | 1,137 / 4,753 (23.92) | 1,817 / 7,555 (24.05) | 1,581 / 6,433 (24.58) | 1,190 / 4,921 (24.18) | 9,854 / 40,832 (24.13) |
| Total | 317,241 / 1,564,912 (20.27) | 71,592 / 341,327 (20.97) | 74,526 / 362,650 (20.55) | 52,074 / 251,732 (20.69) | 58,089 / 271,630 (21.39) | 90,117 / 425,058 (21.20) | 81,299 / 367,232 (22.14) | 58,475 / 274,859 (21.27) | 486,172 / 2,294,488 (21.19) |

COVID-19, coronavirus disease 2019.

^a^Proportion of Cesarean sections = number of Cesarean sections / number of live births.
