# Supplementary material for: Changes in proportions of Cesarean section before and during the COVID‐19 pandemic in Japan
Source: J Obstet Gynaecol Res. 2025 Jul 10;51(7):e16370. doi: 10.1111/jog.16370 (PMC12242368; doi:10.1111/jog.16370)
Supplement: Supplementary file 4 — File S4. Proportion of COVID‐19‐positive Cesarean sections (including suspicious flag) in each prefecture during the COVID‐19 pandemic. [file JOG-51-0-s005.docx]

**File S4.** Proportion of COVID-19-positive Cesarean sections (including suspicious flag) in each prefecture during the COVID-19 pandemic

| **Prefecture** | **Number of COVID-19-positive Cesarean sections / number of births (proportion of COVID-19-positive Cesarean sections^a^, %)** | | | | | | | | |
| --- | --- | --- | --- | --- | --- | --- | --- | --- | --- |
|  | **Pre-COVID-19 period** | **COVID-19 period** | | | | | | | |
|  |  | **Wave 1** | **Wave 2** | **Wave 3** | **Wave 4** | **Wave 5** | **Wave 6** | **Wave 7** | **Wave 1 to 7** |
|  | April 2018 to December 2019 | January to May 2020 | June to October 2020 | November 2020 to February 2021 | March to June 2021 | July to December 2021 | January to June 2022 | July to October 2022 | January 2020 to October 2022 |
| Hokkaido | 0 / 55,830 (0.00) | 0 / 12,205 (0.00) | 1–9 / 12,605  (0.01–0.07) | 10­–20 / 8,841  (0.11–0.23)^b^ | 52 / 9,789 (0.53) | 115 / 14,844 (0.77) | 110 / 12,885 (0.85) | 106 / 9,246 (1.15) | 409 / 80,415 (0.51) |
| Aomori | 0 / 13,034 (0.00) | 0 / 2,839 (0.00) | 0 / 2,964 (0.00) | 0 / 1,986 (0.00) | 1–9 / 2,247 (0.04–0.40) | 1–9 / 3,314 (0.03–0.27) | 40 / 2,954 (1.35) | 28 / 2058 (1.36) | 85 / 18,362 (0.46) |
| Iwate | 0 / 12,794 (0.00) | 0 / 2,760 (0.00) | 1–9 / 2,898  (0.03–0.31) | 1–9 / 2,015 (0.05–0.45) | 1–9 / 2,145 (0.05–0.42) | 1–9 / 3,372  (0.03–0.27) | 1–9 / 2,819 (0.04–0.32) | 1–9 / 2,003 (0.05–0.45) | 20 / 18,012 (0.11) |
| Miyagi | 0 / 27,307 (0.00) | 0 / 5,914 (0.00) | 1–9 / 6,235  (0.02–0.14) | 1–9 / 4,324 (0.02–0.21) | 47 / 4,681 (1.00) | 70 / 7,087 (0.99) | 109 / 6,265 (1.74) | 84 / 4,499 (1.87) | 315 / 3,9005 (0.81) |
| Akita | 0 / 8,529 (0.00) | 0 / 1,826 (0.00) | 0 / 1,949 (0.00) | 1–9 / 1,356 (0.07–0.66) | 0 / 1,505 (0.00) | 1–9 / 2,198 (0.05–0.41) | 1–9 / 1,983 (0.05–0.45) | 1–9 / 1,354 (0.07–0.66) | 13 / 12,171 (0.11) |
| Yamagata | 0 / 11,726 (0.00) | 0 / 2,562 (0.00) | 0 / 2,689 (0.00) | 1–9 / 1,779 (0.06–0.51) | 0 / 1,976 (0.00) | 11 / 3,109 (0.35) | 13 / 2,661 (0.49) | 1–9 / 2,058 (0.05–0.44) | 29 / 16,834 (0.17) |
| Fukushima | 0 / 21,197 (0.00) | 0 / 4,451 (0.00) | 1–9 / 4,881 (0.02–0.18) | 1–9 / 3,422 (0.03–0.26) | 18 / 3,583 (0.50) | 20 / 5,527 (0.36) | 66 / 4,666 (1.41) | 32 / 3,410 (0.94) | 139 / 29,940 (0.46) |
| Ibaraki | 0 / 32,533 (0.00) | 0 / 7237 (0.00) | 14 / 7,311 (0.19) | 16 / 5,197 (0.31) | 29 / 5,623 (0.52) | 39 / 8,523 (0.46) | 57 / 7,685 (0.74) | 55 / 5,628 (0.98) | 210 / 47,204 (0.44) |
| Tochigi | 0 / 22,953 (0.00) | 0 / 4,824 (0.00) | 1–9 / 5,113  (0.02–0.18) | 1–9 / 3,573 (0.03–0.25) | 1–9 / 3,800 (0.03–0.24) | 1–9 / 5,971  (0.02–0.15) | 27 / 5,056 (0.53) | 23 / 3,731 (0.62) | 80 / 32,068 (0.25) |
| Gunma | 0 / 21,748 (0.00) | 0 / 4,791 (0.00) | 1–9 / 4,971  (0.02–0.18) | 1–9 / 3,475 (0.03–0.26) | 40 / 3,790 (1.06) | 108 / 5,869 (1.84) | 225 / 5,041 (4.46) | 198 / 3,861 (5.13) | 577 / 31,798 (1.81) |
| Saitama | 0 / 87,654 (0.00) | 1–9 / 19,274 (0.01–0.05) | 30–40 / 20,350 (0.15–0.20)^b^ | 40 / 13,984 (0.29) | 111 / 15,060 (0.74) | 383 / 24,083 (1.59) | 479 / 20,547 (2.33) | 401 / 15,577 (2.57) | 1,450 / 128,875 (1.13) |
| Chiba | 0 / 74,044 (0.00) | 1–9 / 16,336 (0.01–0.06) | 1–9 / 17,293  (0.01–0.05) | 16 / 11,861 (0.13) | 44 / 12,820 (0.34) | 71 / 20,283 (0.35) | 180 / 17,643 (1.02) | 107 / 13,305 (0.8) | 421 / 109,541 (0.38) |
| Tokyo | 0 / 184,201 (0.00) | 1–9 / 40,407 (0.00–0.02) | 110–120 / 43,407 (0.25–0.28)^b^ | 175 / 28,550 (0.61) | 303 / 32,195 (0.94) | 443 / 50,504 (0.88) | 540 / 42,917 (1.26) | 438 / 32,942 (1.33) | 2,023 / 270,922 (0.75) |
| Kanagawa | 0 / 113,795 (0) | 1–9 / 24,653 (0.00–0.04) | 10–20 / 26,327 (0.04–0.08)^b^ | 38 / 17,900 (0.21) | 98 / 19,579 (0.50) | 215 / 31,242 (0.69) | 303 / 26,747 (1.13) | 227 / 20,419 (1.11) | 902 / 166,867 (0.54) |
| Niigata | 0 / 24,529 (0.00) | 0 / 5,239 (0.00) | 1–9 / 5,673  (0.02–0.16) | 1–9 / 3,963 (0.03–0.23) | 1–9 / 4,216 (0.02–0.21) | 1–9 / 6,497 (0.02–0.14) | 28 / 5,714 (0.49) | 15 / 4,206 (0.36) | 57 / 35,508 (0.16) |
| Toyama | 0 / 11,775 (0.00) | 0 / 2,508 (0.00) | 0 / 2,730 (0.00) | 1–9 / 1,853 (0.05–0.49) | 1–9 / 2,060 (0.05–0.44) | 1–9 / 3,181  (0.03–0.28) | 1–9 / 2,940  (0.03–0.31) | 15 / 2,112 (0.71) | 34 / 17,384 (0.20) |
| Ishikawa | 0 / 14,119 (0.00) | 1–9 / 3,165 (0.03–0.28) | 0 / 3,316 (0.00) | 1–9 / 2,301 (0.04–0.39) | 1–9 / 2,384 (0.04–0.38) | 1–9 / 3,803  (0.03–0.24) | 1–9 / 3,442 (0.03–0.26) | 14 / 2,454 (0.57) | 37 / 20,865 (0.18) |
| Fukui | 0 / 9,776 (0.00) | 0 / 2,179 (0.00) | 0 / 2,254 (0.00) | 1–9 / 1,587 (0.06–0.57) | 1–9 / 1,765 (0.06–0.51) | 1–9 / 2,751  (0.04–0.33) | 1–9 / 2,232 (0.04–0.4) | 18 / 1806 (1.00) | 27 / 14,574 (0.19) |
| Yamanashi | 0 / 9,400 (0.00) | 0 / 2,060 (0.00) | 1–9 / 2,283  (0.04–0.39) | 1–9 / 1,532 (0.07–0.59) | 1–9 / 1,632 (0.06–0.55) | 12 / 2,642 (0.45) | 38 / 2,314 (1.64) | 29 / 1,693 (1.71) | 93 / 14,156 (0.66) |
| Nagano | 0 / 24,382 (0.00) | 0 / 5,080 (0.00) | 0 / 5,598 (0.00) | 1–9 / 3,888 (0.03–0.23) | 0 / 4,173 (0.00) | 10–20 / 6,637 (0.15–0.30)^b^ | 36 / 5,718 (0.63) | 39 / 4,315 (0.9) | 87 / 35,409 (0.25) |
| Gifu | 0 / 23291 (0.00) | 1–9 / 4,901 (0.02–0.18) | 1–9 / 5,162  (0.02–0.17) | 0 / 3,715 (0.00) | 1–9 / 3,858 (0.03–0.23) | 11 / 6,186 (0.18) | 22 / 5,297 (0.42) | 30 / 3,898 (0.77) | 70 / 33,017 (0.21) |
| Shizuoka | 0 / 42,959 (0.00) | 0 / 8,773 (0.00) | 12 / 9,790 (0.12) | 35 / 6,711 (0.52) | 76 / 7,249 (1.05) | 101 / 11,545 (0.87) | 122 / 9,592 (1.27) | 138 / 7,304 (1.89) | 484 / 60,964 (0.79) |
| Aich | 0 / 103,546 (0.00) | 0 / 22,511 (0.00) | 29 / 24,066 (0.12) | 34 / 17,087 (0.2) | 49 / 17,819 (0.27) | 79 / 28,048 (0.28) | 175 / 24,426 (0.72) | 210 / 18,229 (1.15) | 576 / 152,186 (0.38) |
| Mie | 0 / 21,351 (0.00) | 0 / 4,507 (0.00) | 0 / 4,761 (0.00) | 1–9 / 3,372 (0.03–0.27) | 1–9 / 3,581 (0.03–0.25) | 19 / 5,898 (0.32) | 26 / 5,017 (0.52) | 43 / 3,581 (1.2) | 98 / 30,717 (0.32) |
| Shiga | 0 / 19,460 (0.00) | 0 / 4,132 (0.00) | 1–9 / 4,537  (0.02–0.20) | 1–9 / 3,118 (0.03–0.29) | 24 / 3,344 (0.72) | 127 / 5,435 (2.34) | 110 / 4,448 (2.47) | 79 / 3,559 (2.22) | 353 / 28,573 (1.24) |
| Kyoto | 0 / 30,507 (0.00) | 0 / 6,639 (0.00) | 1–9 / 7,102 (0.01–0.13) | 1–9 / 4,961 (0.02–0.18) | 14 / 5,402 (0.26) | 48 / 8,154 (0.59) | 95 / 7,217 (1.32) | 77 / 5,386 (1.43) | 244 / 44,861 (0.54) |
| Osaka | 0 / 112,272 (0.00) | 1–9 / 25,226 (0.00–0.04) | 1–9 / 26,698  (0.00–0.03) | 25 / 18,570 (0.13) | 62 / 19,917 (0.31) | 123 / 31,246 (0.39) | 166 / 27,239 (0.61) | 132 / 20,531 (0.64) | 516 / 169,427 (0.30) |
| Hyogo | 0 / 68,340 (0.00) | 1–9 / 15,085 (0.01–0.06) | 1–9 / 15,935  (0.01–0.06) | 11 / 10,933 (0.10) | 22 / 11,796 (0.19) | 79 / 18,784 (0.42) | 183 / 16,144 (1.13) | 135 / 11,889 (1.14) | 435 / 100,566 (0.43) |
| Nara | 0 / 15,179 (0.00) | 0 / 3,168 (0.00) | 0 / 3,310 (0.00) | 1–9 / 2,367 (0.04–0.38) | 1–9 / 2,606 (0.04–0.35) | 1–9 / 4,131  (0.02–0.22) | 20 / 3,437 (0.58) | 29 / 2,593 (1.12) | 60 / 21,612 (0.28) |
| Wakayama | 0 / 10,410 (0.00) | 0 / 2,363 (0.00) | 1–9 / 2,434  (0.04–0.37) | 0 / 1,771 (0.00) | 0 / 1,872 (0.00) | 1–9 / 2,805  (0.04–0.32) | 1–9 / 2,480 (0.04–0.36) | 21 / 1,866 (1.13) | 36 / 15,591 (0.23) |
| Tottori | 0 / 7,166 (0.00) | 0 / 1,522 (0.00) | 1–9 / 1,630  (0.06–0.55) | 1–9 / 1,171 (0.09–0.77) | 1–9 / 1,250 (0.08–0.72) | 12 / 1,918 (0.63) | 27 / 1,785 (1.51) | 13 / 1,319 (0.99) | 65 / 10,595 (0.61) |
| Shimane | 0 / 8,259 (0.00) | 0 / 1,763 (0.00) | 0 / 1,963 (0.00) | 0 / 1,370 (0.00) | 1–9 / 1,458 (0.07–0.62) | 1–9 / 2,334  (0.04–0.39) | 12 / 1,982 (0.61) | 18 / 1,461 (1.23) | 38 / 12,331 (0.31) |
| Okayama | 0 / 24,722 (0.00) | 0 / 5,507 (0.00) | 0 / 5,895 (0.00) | 1–9 / 4,117 (0.02–0.22) | 14 / 4,342 (0.32) | 10­–20 / 6,767 (0.14–0.30)^b^ | 42 / 5,973 (0.70) | 34 / 4,416 (0.77) | 103 / 37,017 (0.28) |
| Hiroshima | 0 / 36,224 (0.00) | 0 / 7,782 (0.00) | 0 / 8,404 (0.00) | 1–9 / 5,958 (0.02–0.15) | 1–9 / 6,317 (0.02–0.14) | 36 / 9,780 (0.37) | 76 / 8,314 (0.91) | 70 / 6,434 (1.09) | 193 / 52,989 (0.36) |
| Yamaguchi | 0 / 15,581 (0.00) | 0 / 3,361 (0.00) | 1–9 / 3,533  (0.03–0.25) | 1–9 / 2,487 (0.04–0.36) | 1–9 / 2,658 (0.04–0.34) | 1–9 / 4,141  (0.02–0.22) | 57 / 3,704 (1.54) | 69 / 2,797 (2.47) | 143 / 22,681 (0.63) |
| Tokushima | 0 / 8,323 (0.00) | 0 / 1,828 (0.00) | 0 / 1,965 (0.00) | 0 / 1,394 (0.00) | 1–9 / 1,436 (0.07–0.63) | 1–9 / 2,235  (0.04–0.40) | 1–9 / 1,976 (0.05–0.46) | 14 / 1,490 (0.94) | 23 / 12,324 (0.19) |
| Kagawa | 0 / 11,836 (0.00) | 0 / 2,535 (0.00) | 1–9 / 2,635  (0.04–0.34) | 1–9 / 1,852 (0.05–0.49) | 1–9 / 2,162 (0.05–0.42) | 12 / 3,218 (0.37) | 22 / 2,780 (0.79) | 22 / 2,006 (1.1) | 65 / 17,188 (0.38) |
| Ehime | 0 / 15,546 (0.00) | 0 / 3,339 (0.00) | 1–9 / 3,422  (0.03–0.26) | 0 / 2,528 (0.00) | 1–9 / 2,743 (0.04–0.33) | 0 / 4,081 (0.00) | 1–9 / 3,532 (0.03–0.25) | 1–9 / 2,717 (0.04–0.33) | 21 / 22,362 (0.09) |
| Kochi | 0 / 7,687 (0.00) | 0 / 1,726 (0.00) | 0 / 1,663 (0.00) | 0 / 1,321 (0.00) | 1–9 / 1,381 (0.07–0.65) | 1–9 / 2,081  (0.05–0.43) | 1–9 / 1,763 (0.06–0.51) | 11 / 1,335 (0.82) | 22 / 11,270 (0.20) |
| Fukuoka | 0 / 71,480 (0.00) | 1–9 / 15,970 (0.01–0.06) | 92 / 16,696 (0.55) | 80–90 / 11,788  (0.68–0.76)^b^ | 149 / 12,635 (1.18) | 178 / 19,417 (0.92) | 196 / 17,293 (1.13) | 146 / 12,752 (1.14) | 847 / 106,551 (0.79) |
| Saga | 0 / 11,176 (0.00) | 0 / 2,465 (0) | 0 / 2,591 (0.00) | 0 / 1,820 (0.00) | 0 / 1,970 (0.00) | 1–9 / 3,011 (0.03–0.30) | 1–9 / 2,705 (0.04–0.33) | 1–9 / 1,947 (0.05–0.46) | 20 / 16,509 (0.12) |
| Nagasaki | 0 / 17,251 (0.00) | 0 / 3,763 (0.00) | 1–9 / 3,918 (0.03–0.23) | 1–9 / 2,789 (0.04–0.32) | 15 / 3,120 (0.48) | 12 / 4,454 (0.27) | 27 / 3,983 (0.68) | 25 / 2,982 (0.84) | 91 / 25,009 (0.36) |
| Kumamoto | 0 / 24,135 (0.00) | 0 / 5,344 (0.00) | 1–9 / 5,581  (0.02–0.16) | 1–9 / 4,035 (0.02–0.22) | 1–9 / 4,180 (0.02–0.22) | 15 / 6,541 (0.23) | 42 / 5,786 (0.73) | 59 / 4,191 (1.41) | 123 / 35,658 (0.34) |
| Oita | 0 / 13,780 (0.00) | 0 / 3,119 (0.00) | 0 / 3,270 (0.00) | 1–9 / 2,305 (0.04–0.39) | 1–9 / 2,469 (0.04–0.36) | 1–9 / 3,746  (0.03–0.24) | 13 / 3,284 (0.40) | 1–9 / 2,415 (0.04–0.37) | 34 / 20,608 (0.16) |
| Miyazaki | 0 / 14,461 (0.00) | 0 / 3,195 (0.00) | 1–9 / 3,241  (0.03–0.28) | 10–20 / 2,418  (0.41–0.83)^b^ | 12 / 2,511 (0.48) | 28 / 3,944 (0.71) | 30 / 3,415 (0.88) | 24 / 2,514 (0.95) | 109 / 21,238 (0.51) |
| Kagoshima | 0 / 21,785 (0.00) | 0 / 4,591 (0.00) | 11 / 5,028 (0.22) | 18 / 3,692 (0.49) | 21 / 3,778 (0.56) | 86 / 6,166 (1.39) | 129 / 4,998 (2.58) | 132 / 3,649 (3.62) | 397 / 31,902 (1.24) |
| Okinawa | 0 / 26,859 (0.00) | 0 / 5,902 (0.00) | 1–9 / 6,573  (0.02–0.14) | 10–20 / 4,695  (0.21–0.43)^b^ | 12 / 4,753 (0.25) | 33 / 7,555 (0.44) | 98 / 6,433 (1.52) | 56 / 4,921 (1.14) | 217 / 40,832 (0.53) |
| Total | 0 / 317,241 (0.00) | 21 / 341,327 (0.01) | 395 / 362,650 (0.11) | 619 / 251,732 (0.25) | 1,291/ 271,630 (0.48) | 2,593 / 425,058 (0.61) | 4,018 / 367,232 (1.09) | 3,454 / 274,859 (1.26) | 12,391 / 2,294,488 (0.54) |

COVID-19, coronavirus disease 2019.

As a rule regarding the use of National Database of Health Insurance Claims and Specific Health Checkup of Japan, values of 1 to 9 cannot be disclosed.

^a^Proportion of COVID-19-positive Cesarean sections = number of COVID-19-positive Cesarean sections / number of live births. Calculations were made for cases including the suspicious flags.

^b^To ensure that the number in the cell with a value from 1 to 9 in the same row cannot be derived from the total, this number is shown as a range rather than the actual number.
