# Supplementary material for: Changes in proportions of Cesarean section before and during the COVID‐19 pandemic in Japan
Source: J Obstet Gynaecol Res. 2025 Jul 10;51(7):e16370. doi: 10.1111/jog.16370 (PMC12242368; doi:10.1111/jog.16370)
Supplement: Supplementary file 6 — File S6. Difference in the proportion of Cesarean section, COVID‐19 incidence, the population density of inhabitable land area, the number of obstetricians and gynecologists per 100,000 women aged 15–49 years, and the number of births per obstetrician/gynecologist by prefecture. [file JOG-51-0-s006.docx]

**File S6.** Difference in the proportion of Cesarean section, COVID-19 incidence, the population density of inhabitable land area, the number of obstetricians and gynecologists per 100,000 women aged 15 to 49 years, and the number of births per obstetrician/gynecologist by prefecture

|  | Difference in proportion of Cesarean section, %^a^ | COVID-19 incidence, n (per 1 person)^b^ | Population density of inhabitable land area, n (per 1 km^2^ of inhabitable area)^c^ | Number of obstetricians and gynecologists, n (per 100,000 women aged 15 to 49 years)^d^ | Number of births, n (per obstetrician/ gynecologist)^e^ |
| --- | --- | --- | --- | --- | --- |
| Hokkaido | 1.045 | 0.157 | 231.32 | 41.133 | 70.459 |
| Aomori | 0.116 | 0.140 | 381.30 | 40.3667 | 75.051 |
| Iwate | 1.754 | 0.100 | 324.06 | 45.100 | 67.612 |
| Miyagi | 1.843 | 0.124 | 725.06 | 47.200 | 63.481 |
| Akita | -1.236 | 0.118 | 297.48 | 62.833 | 46.370 |
| Yamagata | 1.845 | 0.112 | 370.82 | 48.633 | 66.792 |
| Fukushima | -0.267 | 0.112 | 433.42 | 43.533 | 77.635 |
| Ibaraki | 0.832 | 0.138 | 730.52 | 37.467 | 82.974 |
| Tochigi | 2.274 | 0.122 | 644.82 | 42.300 | 75.411 |
| Gunma | -0.551 | 0.134 | 852.14 | 45.367 | 69.164 |
| Saitama | 0.900 | 0.174 | 2826.92 | 32.400 | 95.084 |
| Chiba | 1.240 | 0.158 | 1772.06 | 36.400 | 85.720 |
| Tokyo | 1.232 | 0.241 | 9841.94 | 55.967 | 52.837 |
| Kanagawa | 0.721 | 0.167 | 6263.98 | 40.400 | 76.511 |
| Niigata | 1.196 | 0.115 | 484.20 | 38.800 | 82.172 |
| Toyama | 0.497 | 0.136 | 561.18 | 49.167 | 69.007 |
| Ishikawa | 1.635 | 0.156 | 812.30 | 48.233 | 70.939 |
| Fukui | 0.684 | 0.162 | 710.64 | 48.167 | 74.764 |
| Yamanashi | 1.081 | 0.130 | 850.02 | 56.133 | 61.702 |
| Nagano | -0.260 | 0.117 | 631.90 | 41.967 | 82.317 |
| Gifu | 0.295 | 0.161 | 893.68 | 46.767 | 69.679 |
| Shizuoka | 1.197 | 0.144 | 1312.62 | 43.767 | 74.850 |
| Aich | 1.255 | 0.194 | 2516.18 | 44.333 | 78.599 |
| Mie | 1.039 | 0.156 | 857.76 | 45.833 | 71.569 |
| Shiga | 0.799 | 0.178 | 1084.52 | 40.600 | 87.767 |
| Kyoto | 1.486 | 0.193 | 2190.52 | 47.567 | 61.547 |
| Osaka | 0.689 | 0.247 | 6618.90 | 49.833 | 64.405 |
| Hyogo | 0.531 | 0.189 | 1966.80 | 45.333 | 72.145 |
| Nara | 2.136 | 0.176 | 1549.22 | 45.767 | 67.605 |
| Wakayama | -1.195 | 0.150 | 823.32 | 58.233 | 59.079 |
| Tottori | 1.137 | 0.122 | 612.56 | 63.567 | 60.204 |
| Shimane | 1.243 | 0.135 | 523.04 | 61.333 | 61.710 |
| Okayama | 1.058 | 0.156 | 847.62 | 51.867 | 69.250 |
| Hiroshima | 0.862 | 0.169 | 1213.52 | 41.000 | 86.616 |
| Yamaguchi | 0.918 | 0.132 | 783.86 | 48.033 | 72.137 |
| Tokushima | -0.371 | 0.137 | 710.12 | 65.533 | 52.505 |
| Kagawa | 1.397 | 0.161 | 944.48 | 48.833 | 70.146 |
| Ehime | 1.080 | 0.129 | 798.72 | 51.600 | 65.595 |
| Kochi | 0.437 | 0.150 | 595.22 | 53.067 | 65.042 |
| Fukuoka | 0.772 | 0.215 | 1856.00 | 45.633 | 76.975 |
| Saga | 0.088 | 0.201 | 607.70 | 47.900 | 76.822 |
| Nagasaki | 0.635 | 0.166 | 785.28 | 60.933 | 63.827 |
| Kumamoto | 1.630 | 0.198 | 628.40 | 45.233 | 84.931 |
| Oita | 0.409 | 0.165 | 625.84 | 48.233 | 73.301 |
| Miyazaki | 0.010 | 0.191 | 572.98 | 52.167 | 74.817 |
| Kagoshima | 1.716 | 0.194 | 481.74 | 57.033 | 70.734 |
| Okinawa | 0.990 | 0.352 | 1280.82 | 50.733 | 93.517 |

COVID-19, coronavirus disease 2019.

^a^The difference in the proportion of Cesarean sections between the pre-COVID-19 and COVID-19 pandemic periods was calculated by the proportion of Cesarean section in the COVID-19 period minus the proportion of Cesarean section in the pre-COVID-19 pandemic period for each prefecture.

^b^The COVID-19 incidence for each prefecture was calculated by the number of COVID-19-positive cases for each prefecture from April 1, 2020 to October 31, 2022 divided by the Japanese population for each prefecture on October 1, 2020.

^c^The population density of inhabitable area was calculated by averaging the annual data on the population density of inhabitable land area from 2018 to 2022.

^d^The number of obstetricians and gynecologists per 100,000 women aged 15 to 49 was calculated by the mean of each data of the number of physicians with board certification in obstetrics and gynecology per 100,000 women aged 15 to 49 years in 2018, 2020, and 2022.

^e^The number of births per obstetrician/gynecologist was calculated as the average of the number of births in the fascial years 2018, 2020, and 2022 (April to March of the following year) divided by the total number of physicians with board certification in obstetrics and gynecology in 2018, 2020, and 2022.
